# A neuroprotective target in the shadows: a scoping review of pharmacological gaps in σ1R agonists for glutamate-induced neurotoxicity

**DOI:** 10.3389/fphar.2026.1829506

**Published:** 2026-09-07

**Authors:** Alexa Q. Xiang, Madison Thalmann, Izna Khanna, Shrishti Harish, Mihir Khanna, Aarush Kulkarni, Leqing Chen

**Affiliations:** 1 Academy Program, The Ohio State University, Columbus, OH, United States; 2 Department of Psychology, Kean University, Union, NJ, United States; 3 Saratoga High School, Saratoga, CA, United States; 4 Mountain House High School, Mountain House, CA, United States; 5 Wissahickon High School, Ambler, PA, United States; 6 Heritage High School, Wake Forest, NC, United States; 7 Lowell High School, San Francisco, CA, United States

**Keywords:** agonists, glutamate toxicity, neuroprotection, sigma-1 receptor, σ1R

## Abstract

**Introduction:**

Glutamate‐induced neurotoxicity is a shared pathological mechanism across neurodegenerative and neuropsychiatric disorders, but the mechanistic contributions of the sigma‐1 receptor remain unsolved and underappreciated.

**Methods:**

This scoping review focuses on the pharmacological gaps in σ1R agonists by synthesizing mechanistic and bibliometric trends across the most influential literature in the field currently. To map how σ1R agonism has historically been thought of and where mechanistic blind spots still persist, we analyzed the top 100 most cited articles from Web of Science, Scopus, and Google Scholar. This approach was grounded in established scientometric methodology for capturing dominant scientific findings and research trajectories, using a PICO‐structured framework and evaluating how σ1R agonists influence and modulate glutamate‐induced excitotoxicity compared with non‐σ1R interventions, focusing on neuroprotection, calcium regulation, mitochondrial stability, and translational readiness.

**Results:**

Although several agonists like PRE‐084, pridopidine, SA4503 and other neurosteroids show mechanistic convergence on ER-mitochondria signaling and calcium homeostasis, citation patterns reveal substantial gaps. These gaps include limited post‐2015 mechanistic studies and preclinical bottlenecks that have impeded clinical translation.

**Discussion:**

Despite convergent mechanisms among several agonists, progress toward clinical translation remains limited by challenges in ligand specificity and model fidelity, and the predominance of preclinical evidence. Addressing these limitations may facilitate the development of σ1R-targeted therapies for neurodegenerative and other neurological disorders characterized by disrupted calcium signaling and glutamatergic dysfunction.

## 1 Introduction

Glutamate toxicity, often referred to as excitotoxicity, is a process in which excessive activation of glutamate receptors induces neuronal damage. It has been widely implicated as a central mechanism in neurodegenerative diseases, including Alzheimer’s disease, amyotrophic lateral sclerosis (ALS), Parkinson’s disease, and Huntington’s disease (Anderson et al., 2005; Antonini et al., 2011; Aren et al., 2011; Dong et al., 2007). In healthy neural networks, glutamate serves as the principal excitatory neurotransmitter by playing a critical role in synaptic transmission, plasticity, learning, and memory (Hayashi et al., 1995; Hayashi and Su, 2004). Its tightly regulated signaling also helps maintain proper calcium homeostasis and support energy metabolism in neurons (Hayashi and Su, 2007; Zheng, 2009).

However, when extracellular glutamate concentrations rise abnormally through impaired reuptake by astrocytes or dysregulation in general, the resulting overstimulation of NMDA and AMPA receptors triggers a cascade of harmful events. These include massive calcium influx, mitochondrial dysfunction, generation of reactive oxygen species, and activation of apoptotic pathways, which cause synaptic loss and progressive neuronal degeneration (Anderson et al., 2005; Chen et al., 2006; Dong et al., 2007; Jiang et al., 2006). This excitotoxic cascade is a common feature across both acute neurological injuries (such as stroke) and chronic neurodegenerative conditions (Allahtavakoli and Jarrott, 2011; Antonini et al., 2011).

The global burden of neurodegenerative diseases is substantial, with over 50 million individuals estimated to suffer worldwide. Glutamate dysregulation has been identified as a key contributing factor across many of these conditions, emphasizing the urgent need for effective therapeutic strategies (Gautam et al., 2023). The sigma-1 receptor (σ1R) has become a main target for neuroprotective intervention because it regulates calcium homeostasis and ER-mitochondrial stress responses, key pathways disrupted during glutamate-induced excitotoxicity (Hayashi and Su, 2007; Maurice and Su, 2009; Hashimoto, 2015). Rather than functioning as a single-pathway intervention, σ1R is a multi-functional regulatory target capable of stabilizing several interconnected mechanisms underlying excitotoxic degeneration simultaneously. This is significant, because glutamate toxicity rarely progresses through one isolated pathway; instead, excessive glutamate signaling initiates overlapping cycles of calcium dysregulation, oxidative stress, mitochondrial collapse, and apoptotic signaling that collectively accelerate neuronal death. By acting upstream of these interconnected events, σ1R activation can reduce the propagation of widespread cellular dysfunction. This is superior to blocking a single downstream effector (Hashimoto, 2015; Hayashi and Su, 2004; Maurice and Lockhart, 1997).

Localized at the interface between the endoplasmic reticulum (ER) and mitochondria, σ1R is a ligand-operated molecular chaperone that regulates calcium signaling to stabilize stress levels and preserves mitochondrial function (Hayashi and Su, 2007; Maurice and Su, 2009). This is significant because the ER-mitochondria interface is a critical regulatory site for neuronal survival during glutamate-induced stress and is also where dysregulated calcium transfer can trigger energy depletion and apoptosis (Hayashi and Su, 2007). Activation of σ1R has been shown to limit pathological calcium accumulation and reduce apoptotic signaling in neuronal models (Chen et al., 2006; Guo et al., 2013); σ1R has the versatility to limit the progression of excitotoxic injury and prevent irreversible mitochondrial failure associated with progressive neurodegeneration (Hayashi et al., 1995). Beyond maintaining cellular homeostasis, σ1R also stabilizes ER-mitochondria communication and enhances neuronal resilience under chronic stress (Maurice and Su, 2009). Its neuroprotective capability extends beyond acute cellular stabilization toward sustaining long-term neuronal resilience under chronic pathological stress (Hayashi and Su, 2007; Zheng, 2009).

Throughout this review, it is important to distinguish between σ1R ligands, which include all receptor-binding compounds, and σ1R agonists, which specifically activate the receptor to initiate downstream protective signaling. σ1R ligands include any compound capable of binding to the sigma-1 receptor, including agonists, antagonists, and other compounds with different pharmacological effects. In contrast, σ1R agonists are a specific group of ligands that activate the receptor and trigger downstream neuroprotective signalling. Because this review focuses on glutamate-induced neurotoxicity, σ1R agonists are the primary focus since receptor activation has been associated with reduced calcium overload, improved mitochondrial function, and decreased neuronal cell death. However, the broader term “σ1R ligand” is used when discussing the overall pharmacological development of the field, as many studies investigate receptor-binding compounds before determining whether they function as agonists or antagonists.

Pharmacological activation of σ1R through selective agonists such as PRE-084, pridopidine, and neurosteroids including dehydroepiandrosterone (DHEA) has demonstrated preliminary effectiveness in preclinical models (Maurice et al., 1999). The neuroprotective effects observed across these compounds suggest that σ1R activation may produce broad downstream stabilization despite differences in ligand structure and upstream signaling pathways. Consistently suggested, σ1R is a convergent therapeutic regulator. These compounds have been associated with reductions in neuronal injury and improvements in cognitive and motor outcomes across disease models, even encompassing retinal injury beyond the most-researched neurodegenerative disorders (Allahtavakoli and Jarrott, 2011; Antonini et al., 2011; Aren et al., 2011; Dun et al., 2007; Francardo et al., 2014; Maggio et al., 2014; Mancuso et al., 2012; Maurice and Goguadze, 2017; Ruscher et al., 2011; Zhang et al., 2019; Zhao et al., 2017).

This distinction is important, because σ1R binding alone does not guarantee neuroprotection and only agonists that activate the receptor’s chaperone function produce downstream protective effects. Instead, activation of the receptor by σ1R agonists is responsible for the signalling pathways that help reduce glutamate-induced excitotoxicity. Therefore, although this review discusses the broader landscape of σ1R ligands, it primarily evaluates evidence supporting σ1R agonists as potential therapeutic agents.

For example, σ1R agonists have demonstrated the capacity to reduce neuronal damage following ischemic injury while simultaneously improving behavioral and cognitive recovery; σ1R’s modulation ability that could influence both cellular survival and functional neurological outcomes. DHEA administration improves memory deficits following ischemia through σ1R stimulation (Yasushi et al., 2015). Progesterone, a steroid hormone, also confers neuroprotection through σ1R, though via distinct upstream signaling cascades compared to DHEA (Cai et al., 2008). However, despite this mechanistic divergence, both steroids ultimately engage σ1R’s chaperone function at the ER-mitochondria interface, suggesting that σ1R projects multiple forms of neurological injury. The ability of these ligands to engage σ1R despite their structural differences makes σ1R a compelling candidate for translational research, especially in contexts where traditional therapeutic approaches have failed to yield meaningful benefits (Hashimoto, 2015; Zhang et al., 2022).

Despite these extensive preclinical investigations, the translation of σ1R-targeted therapies into clinical practice remains remarkably limited. Therapeutic strategies that directly mitigate excitotoxicity are scarce, and little to none have successfully progressed to human trials (Hashimoto, 2015; Zhang et al., 2022). This gap is compounded by several fundamental challenges: incomplete characterization of σ1R ligand selectivity, cross-reactivity with other receptors such as NMDA and α7 nicotinic acetylcholine receptors, inconsistent outcomes across experimental models, and suboptimal pharmacokinetic and pharmacodynamic properties of candidate compounds (Guo et al., 2013; Hayashi and Su, 2004).

Given the heterogeneity of σ1R research and the breadth of experimental contexts in which σ1R agonists have been studied, a scoping review approach was selected as the most appropriate methodology to address the objectives of this work. Unlike systematic reviews that aim to answer narrowly defined questions, scoping reviews are particularly well suited for mapping the extent and nature of evidence across fragmented and multidisciplinary fields and clarifying conceptual and methodological trends. Research on σ1R spans molecular biology, pharmacology, chemical and pharmaceutical engineering, neurodegeneration, psychiatry, and medicinal chemistry, with substantial variability. This diversity necessitates an approach capable of integrating perspectives without restricting inclusion to a single disease model or clinical outcome.

The population of interest encompasses systems studied with *in vitro*/*in vivo* and limited clinical contexts. By exploring these elements, this scoping review aims to clarify the current state of σ1R research, including persistent limitations, and identify priority areas for future investigation. This scoping review’s framework maps the landscape of σ1R-related research alongside mechanistic pathway evaluation (Haddaway et al., 2022; Tricco et al., 2018). This is critical for advancing comprehension of glutamate-mediated neurodegeneration and development of more precise drug delivery methods capable of alleviating the adverse impacts of neurodegeneration, and this section establishes the scientific rationale for the review and explain why σ1R-mediated regulation of glutamate toxicity is the central focus of the work.

## 2 Methods

The PRISMA 2018 statement for scoping reviews was followed (Tricco et al., 2018). The objective of this review was to identify pharmacological and trend-based translational gaps in σ1R agonists within the context of glutamate-induced neurotoxicity. Since the goal was to characterize research trends and dominant mechanistic frameworks instead of evaluating intervention efficacy, we intentionally analyzed the top 100 most cited articles from Web of Science, Scopus, and Google Scholar. Citation-based sampling is a validated bibliometric strategy for mapping influential scientific developments, identifying conceptual blind spots, and characterizing how research fields evolve over time (Bornmann and Daniel, 2008; van Raan, 2019). Highly cited articles can disproportionately shape scientific understanding, guide subsequent experimental design, and define mechanistic assumption so this approach was most appropriate for identifying persistent gaps in σ1R pharmacology. Since bibliometric mapping was significant for identifying how σ1R research has developed over time, the review also examined authorship patterns and global research distribution to contextualize pharmacological and mechanistic gaps within the whole structure of the field.

A modified PICO framework was used to structure the review question and organize the screening process. The population included experimental systems where glutamate-induced excitotoxicity was modeled and the intervention was defined as any pharmacological activation of σ1R evaluated within this context. Comparator conditions consisted of either non-σ1R treatments, σ1R antagonism, or untreated excitotoxic controls. Outcomes were broadly defined to include any neuroprotective or functional measures relevant to excitotoxic injury. This framework made sure that the included studies consistently addressed the central question of how σ1R agonists have been investigated in relation to glutamate toxicity and where mechanistic or translational gaps remain, while allowing the bibliometric component of the review to map how these themes have been represented across influential literature. This scoping review focused on elucidating the role of σ1R agonism in modulating glutamate-induced neurotoxicity and related neuroprotective mechanisms. To capture the most influential and impactful research in this area, we limited our primary datasets to the top 100 most cited articles identified through the Web of Science Core Collection (WoS Core), Google Scholar, and Scopus. Citation count was used as a proxy for scientific influence, allowing us to prioritize studies that have significantly shaped the field; the articles were obtained by sorting each database’s results by most cited to least cited using provided filters, then exporting the metadata of the first 100 studies for each. Not intended as a direct measure of scientific accuracy, this approach was implemented because the objective of review was to analyze the most prevalent research trends and development within σ1R-mediated neuroprotection text, and their associated gaps. However, the citation-based methodology may favor older cited publications while potentially underrepresenting recent, emerging studies. To resolve this problem, multiple databases with different indexing systems were searched using broad and overlapping terminology related to only glutamate toxicity and related pathways in σ1R pharmacology. Screening procedures prioritized scientific relevance in addition to citation indicators, reducing the probability that conclusions were determined by only citation frequency.

Included studies were required to satisfy all of the following criteria.

1. Studies had to address σ1R pharmacology in a substantive way, meaning that receptor function, ligand activity, agonist or antagonist characterization, or receptor-dependent signaling had to be a central focus or element of the study rather than a brief mention.
2. Studies had to meaningfully engage with either glutamate-induced neurotoxicity or mechanistically related pathways that have a casual impact on excitotoxic injury, including calcium homeostasis dysregulation, ER-mitochondria signaling disruption, oxidative stress, or apoptotic cascade activation; this study intends to map both glutamate-induced neurotoxicity in specific and broader pathways in context.
3. Eligible studies had to employ cell-based or animal experimental systems, or present clinically relevant data pertaining to σ1R-mediated neuroprotection, confirming that the evidence base captured both mechanistic granularity and translational context.
4. Only full-text peer-reviewed research articles and review articles available in the English language were considered, as these publication types provide the methodological transparency necessary for accurate and consistent data extraction across the research team.

Studies were excluded if they met any of the following conditions.

1. Articles in which σ1R appeared non-centrally or without evaluation of receptor function, ligand activity, or σ1R-dependent neuroprotection were removed.
2. Studies that did not engage with mechanistic pathways interconnected to glutamate toxicity were excluded, as they did not address the central question of the review.
3. Publication types lacking sufficient methodological detail or formal peer review, including editorials, commentaries, conference abstracts, theses, and preprints, were removed.
4. Full texts not available in English were excluded to maintain consistency in data extraction across the team.
5. Duplicate records appearing across multiple databases were identified through DOI matching and title cross-verification and were removed prior to analysis to prevent the same articles from artificially inflating citation rankings in the dataset.

The search strategy combined a boolean statement keywords about the concepts of interest; “Glutamate Toxicity” or “Glutamate,” and “Neuroprotection,” were paired with “Sigma-1 Receptor” or “σ1R” for specificity. No temporal limits were imposed to capture the historical evolution of research in σ1R. The search strategy was explicitly designed to minimize bias; keyword combinations were standardized across all databases, and boolean operators were used to ensure consistent retrieval logic: ‘Glutamate Toxicity’ OR ‘Glutamate’ AND ‘Neuroprotection’ AND ‘Sigma-1 Receptor’ OR 'σ1R.'

To ensure maximum sensitivity and precision while capturing historical and developmental trends, a deductive process was used to establish the primary search terms based directly on the core research questions. Given the complex nomenclature landscape surrounding the target receptor, which includes its official gene symbol (SIGMAR1) and its systematic chemical name (sigma non-opioid intracellular receptor 1), searching via extended molecular definitions or over-truncated shorthand risked introducing irrelevant non-opioid receptor data. Consequently, the primary search syntax was designed around the two most ubiquitous, internationally recognized structural designations in biochemical literature, “sigma-1 receptor” and “σ1R”. To account for localized shorthand variants used within individual manuscripts, a post-hoc diagnostic validation was performed; authors who utilize colloquial or text-body abbreviations such as “Sig1R” almost universally introduce the receptor by its full standard name or symbol within the title, abstract, or official keywords that were searched across databases to ensure discoverability; furthermore, modern database search engines utilize lemmatization algorithms that automatically map alphanumeric variants to their standardized parent terms. Accordingly, the primary search syntax successfully achieved comprehensive coverage of the target literature.

Among the three databases, Web of Science offered the most structured bibliometric infrastructure, including selective journal indexing and citation-level metadata that enabled co-authorship network analysis (Mongeon and Paul-Hus, 2016). In addition, Scopus offers a broader coverage of pharmacological and biomedical literature than Web of Science, especially throughout European journals, and is similarly validated for bibliometric mapping studies (Falagas et al., 2008). Unlike the other two databases, Google Scholar does not restrict indexing to curated journal lists, which means it surfaces interdisciplinary and less formally indexed literature that would otherwise fall outside the search scope of the review (Haddaway et al., 2015). Critically, all three databases allow results to be sorted by citation count and support structured metadata export, which is essential for citation-ranked bibliometric sampling (Bornmann and Daniel, 2008; van Raan, 2019).

PubMed and EMBASE, while being highly valuable databases for systematic reviews and clinical evidence syntheses, were not included as databases for citation-based sampling in the review because neither platform natively supports sorting search results by citation frequency or provides the standardized citation metadata required for bibliometric network analysis (Falagas et al., 2008; Mongeon and Paul-Hus, 2016). This represents a limitation of the current study, as PubMed and EMBASE index pharmacological and clinical trial literature that may not be entirely captured in the dataset. Future scoping and bibliometric work in this area should seek to incorporate these databases where citation-ranking tools become more accessible, or employ hand-searching strategies to compensate.

Metadata was extracted and exported using Microsoft Excel and BibTeX. Metadata were managed in xlsx/.csv, and .bib formats to support both qualitative and bibliometric analyses. A dataset was created by merging all databases output before analysis, and data consolidation was performed using DOI matching title classification, cross-checking auto, and verifying publication year. Variables extracted for analysis included senior author names, to capture contributions and authorship networks; publication year, to assess historical gaps; article title and source journal for categorization; citation count, as an indicator of influence; author affiliations, to map institutional contributions; and experimental focus. A bibliometric analysis was conducted individually for each database after deduplication; as each database uses unique formats and indexing criteria, analyzing them separately shows comparisons of trends and impacts across different databases. However, results were pooled for thematic grouping and literature review as the nature of σ1R itself does not differ across research containers.

Article screening and data extraction were conducted by a group of 27 research interns, with tasks distributed evenly and following a standardized process, reducing individual reviewer bias. Full text revision was conducted once by the research team, with each research intern completing a standardized extraction form that captured the study’s experimental model, σ1R ligand or agonist type, outcome measures related to neuroprotection or calcium regulation, and key mechanistic findings. Narrative summaries based on these forms were then synthesized. The research team synthesized findings through thematic integration based on these individual article summaries. Articles were grouped into thematic categories and visualizations were used to contextualize and elucidate research trends; these findings are summarized in a comprehensive table in Supplementary Material.

Bibliometric variables, which primarily consisted of author details and institutional affiliations, were visualized in the R programming language using the Biblioshiny package to map co-authorship networks and global research trends. Bar plots and comparative visualizations of research trends were created in Microsoft Excel. Public domain NIH BioArt, BioIcons, Wikimedia Commons images, and bitmap drawings were compiled using Kleki Paint Tool and Google Drawings to illustrate elucidated concepts, including mechanistic structure diagrams, word clouds, interaction networks, and heat maps assessing relative efficacies of σ1R agonist drugs.

While the PCC (population, concept, context) framework is utilized for scoping reviews to maintain a broad conceptual field, this review intentionally adopts a modified PICO (population, intervention, comparator, outcome) framework. As the primary objective is to map specific pharmacological gaps and drug-development bottlenecks for σ1R agonists, a PICO framework addresses the distinct biochemical mechanisms and drug variations that compose the preclinical-to-clinical outcomes, instead of categorizing them into broad “concepts”. By utilizing PICO, we systematically categorize the literature based on distinct intervention types and map possible outcomes and gaps in those outcomes. This identifies precise failure points in the pharmacological pipeline while still assessing trends in scientific development over the efficacy of individual effects themselves, thereby fulfilling the scoping objective in a methodologically rigorous manner.

This study used a mixed-methods approach for the scoping review by utilizing a bibliometric analysis. The general PRISMA-compliant review practice allowed for summarizing and synthesizing evidence on σ1R agonists, glutamate-induced neurotoxicity, neuroprotective mechanisms, and translational challenges across different study types. The bibliometric analysis complemented this by examining publication trends, highly cited studies, leading authors, institutions, and collaboration networks to better understand how the field has developed over time. Combining these two methods was beneficial, because the objective of this study included identifying research trends and gaps in the literature. While the qualitative evidence explained what is currently known about σ1R-mediated neuroprotection, the bibliometric analysis showed where research has been concentrated and where additional investigation is still needed. Together, these approaches provided a broader understanding of both the scientific evidence and the overall development of σ1R research.

The final synthesis emphasized quantitative collective insights and qualitative overarching trends, instead of isolated study findings. Bibliometric findings were interpreted separately from pharmacological conclusions, and citation frequency was not used as evidence of clinical validity. Instead, the results were interpreted as trend-based and mechanistic, providing a structured and integrated understanding of σ1R-mediated neuroprotection. A general review protocol was registered with The Open Science Framework (registration ID: 9fa8x) during the revision stage of this study. Overall, these methods ensure the review captures the most influential but also relevant and informative work on σ1R and glutamate-related neuroprotection.

## 3 Results

**FIGURE 1:**
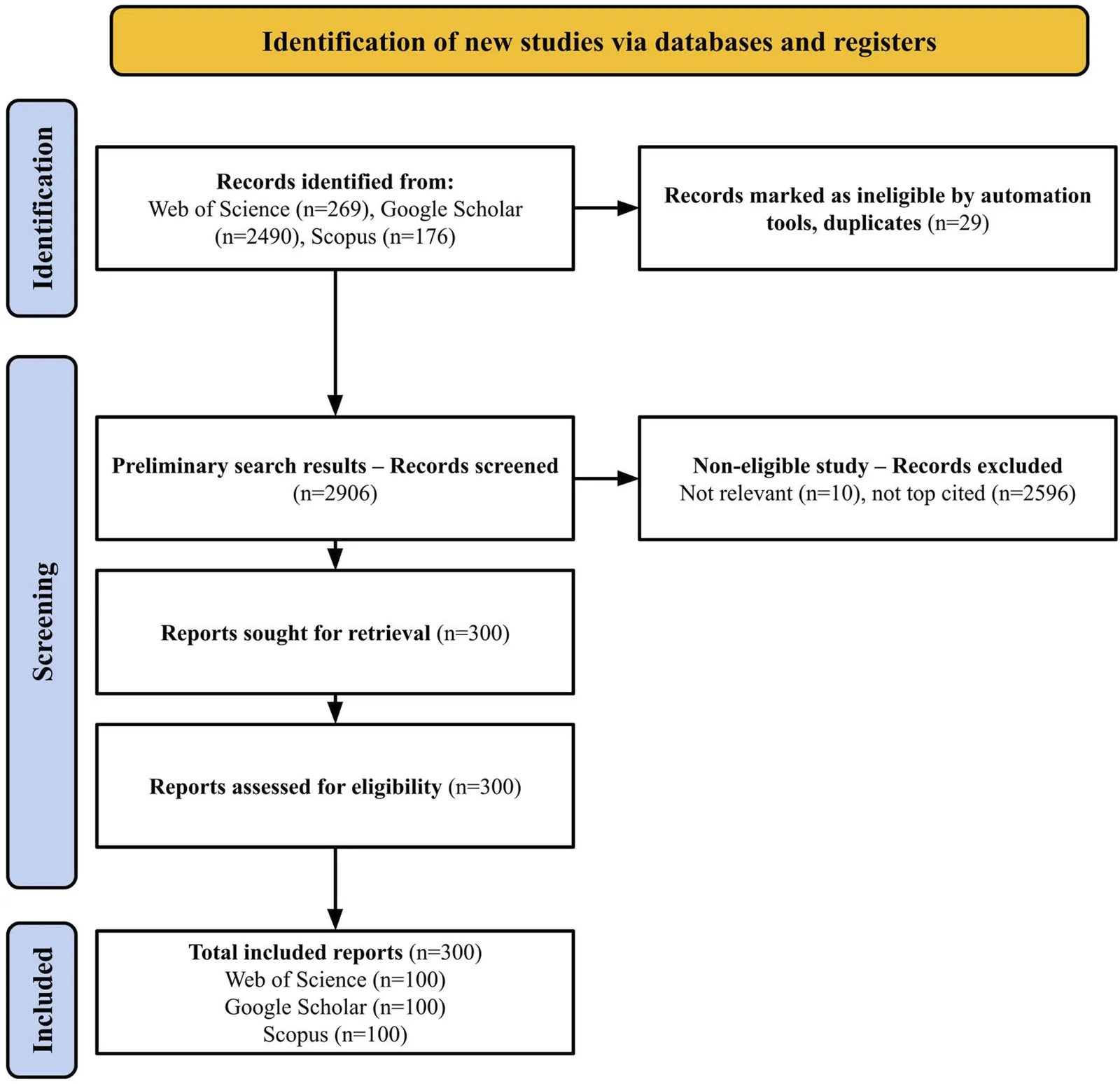
PRISMA flow diagram for study selection

Identified records and screening phases of the literature search are documented in Figure 1.

### 3.1 Global authorship and institutional patterns

**FIGURE 2:**
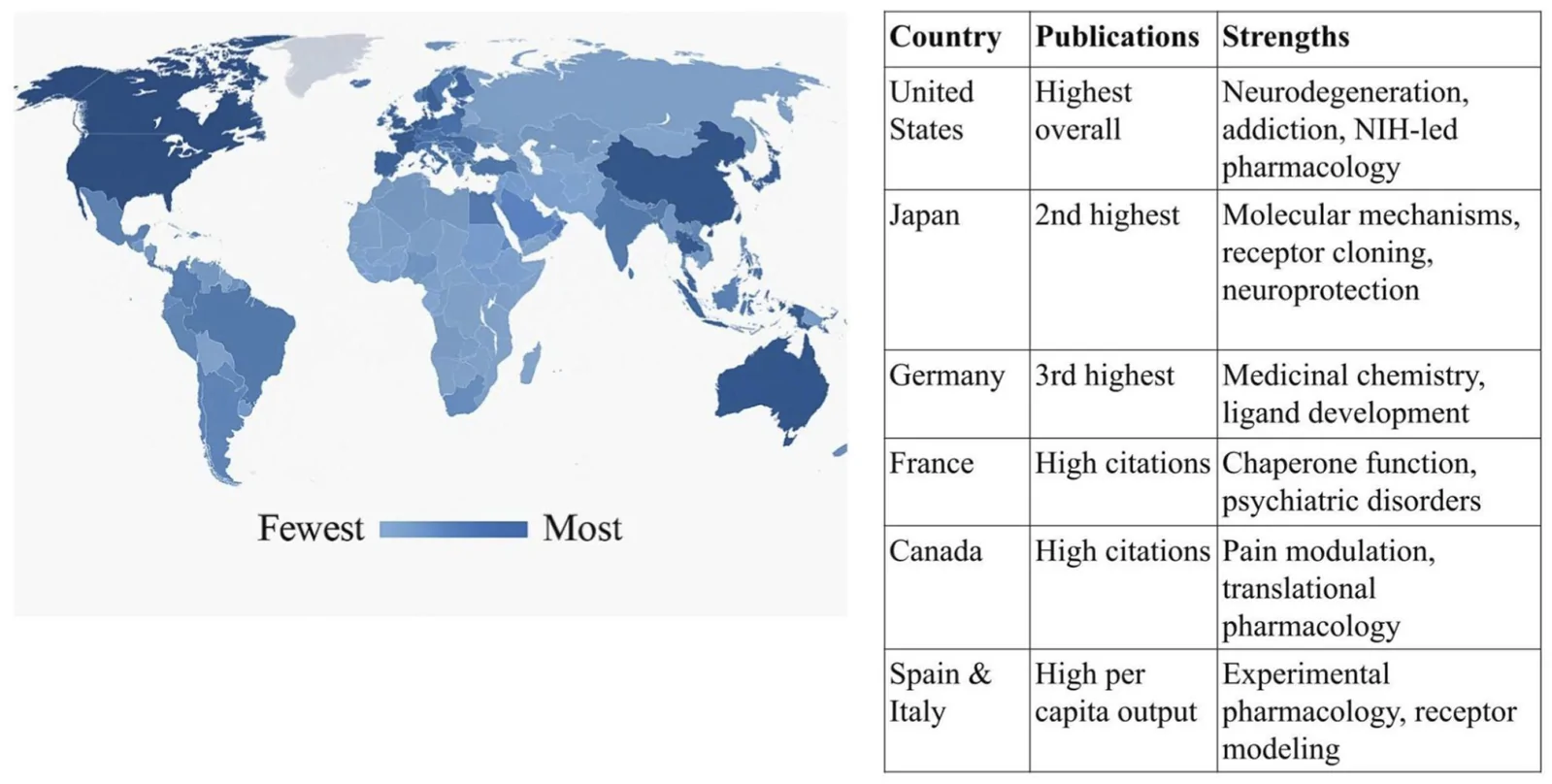
σ1R research by country.

Research on σ1R agonism and glutamate-induced neurotoxicity is globally diverse and it spans disciplines such as neurodegeneration, pharmacology, and medicinal chemistry (Maurice and Su, 2009; Hashimoto, 2015). Senior authors were widely distributed across Europe, North America, and Asia, showing geographically heterogeneous contributions (Chen et al., 2006; Hayashi and Su, 2004; Jiang et al., 2006; Maurice and Lockhart, 1997). Despite this, regions like North America and Western Europe exhibited higher volume of research activity, while parts of Asia, Africa, and Eastern Europe produced fewer publications, as shown in Figure 2 (Guo et al., 2013; Hashimoto, 2015; Romero and Portillo-Salido, 2019; Zheng, 2009).

The United States led in overall publications, with particular strength in neurodegeneration and addiction research (Allahtavakoli and Jarrott, 2011; Zhao et al., 2017). Japan ranked second, with notable contributions in molecular mechanisms and receptor cloning. Germany contributed substantially in medicinal chemistry and ligand development, while France, Canada, Spain, and Italy showed high *per capita* output that focused on psychiatric disorders and experimental receptor pharmacology (Antonini et al., 2011; Aren et al., 2011; Cobos et al., 2008; Hayashi et al., 1995; Hayashi and Su, 2007; Köhler et al., 2012). There is therefore strong evidence of specialization across the global research landscape.

**FIGURE 3:**
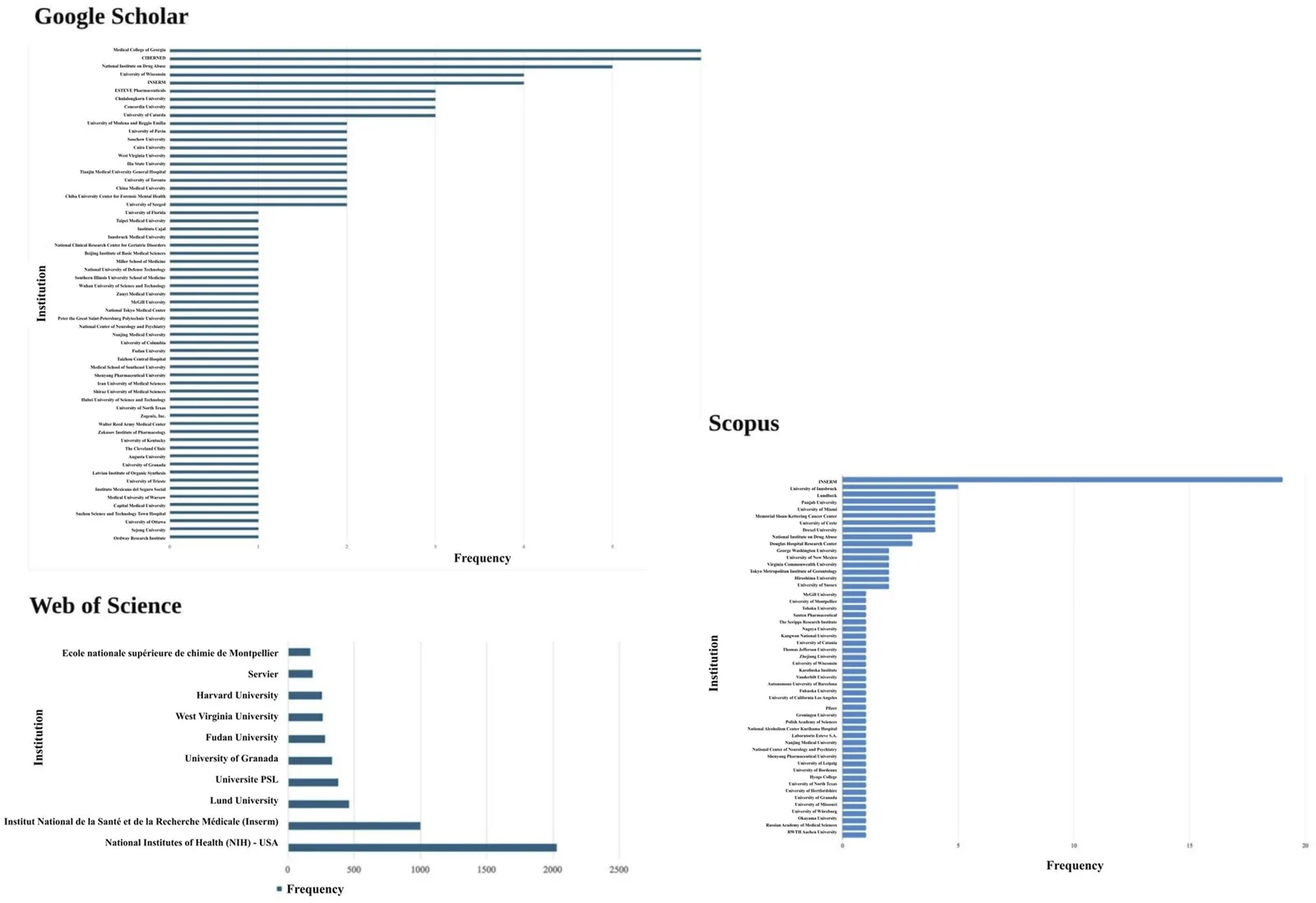
Senior authors’ institutions with multiple publications of the top 100 most cited studies.

**FIGURE 4:**
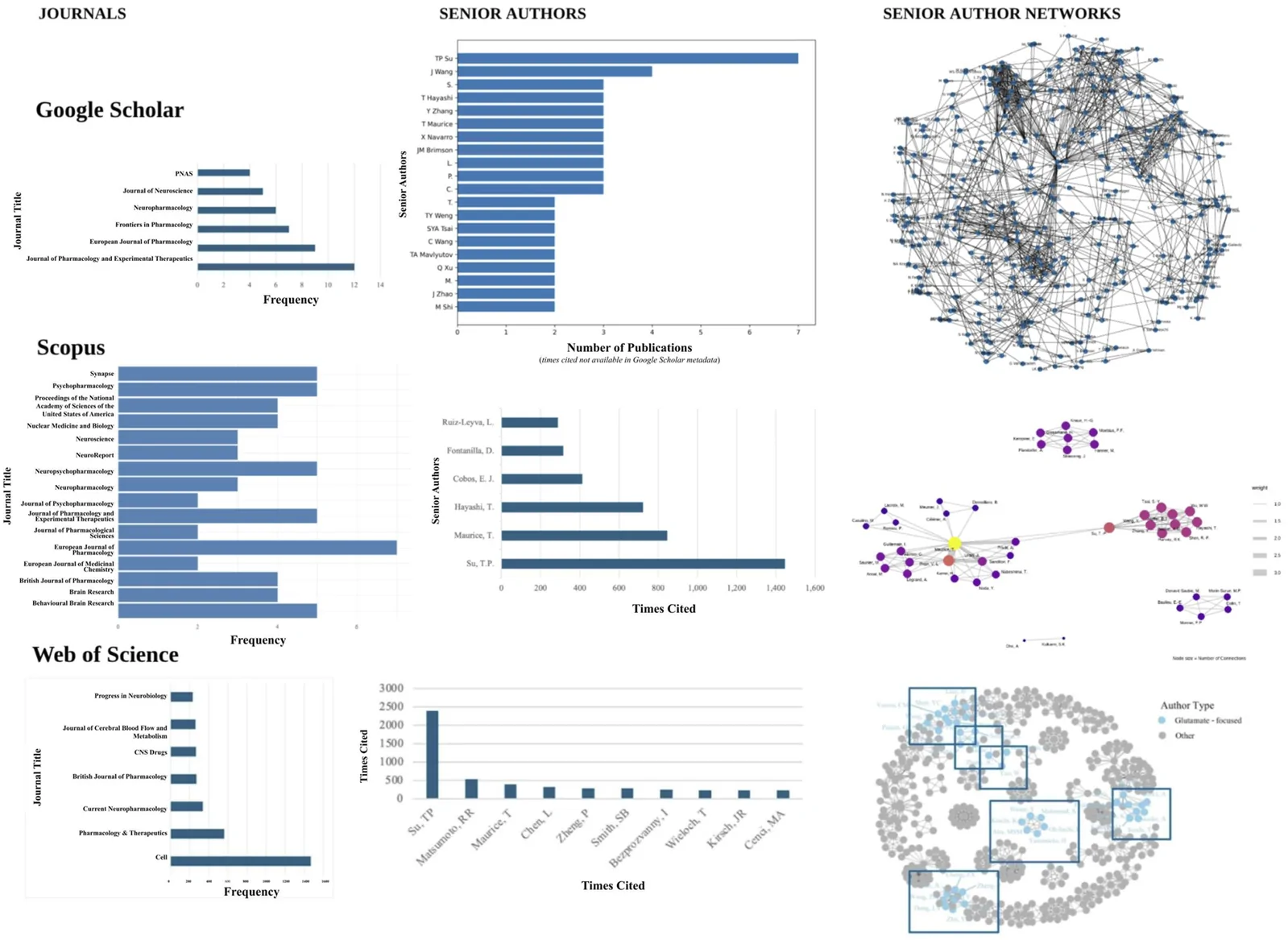
Journals and senior authors with multiple publications of the top 100 most cited studies; co-authorship networks.

In Web of Science, top institutions included the National Institute of Health (NIH) in the United States, Johns Hopkins University, Augusta University, and several leading universities in France, Germany, China, and Japan (Zhang et al., 2019; Zhao et al., 2017). Interestingly, national institutes in Latin America and China occasionally surpassed certain top U.S. universities in citation impact, likely due to targeted research initiatives or growing momentum in the field; this has been shown in Figure 3. A top-heavy distribution of publications and research advancements can be observed when examining similar types of patterns among journals in Web of Science, with CELL far in the lead, as reported in Figure 4 (Hayashi and Su, 2007; Ruscher et al., 2011).

Scopus shows a moderately concentrated distribution across several core pharmacology and neuroscience journals. The European Journal of Pharmacology appears as the most recurrent journal within the dataset. σ1R research in Scopus also appears to be distributed across several journals rather than centralized within one primary publication venue.

When compared to Google Scholar, a slightly stronger clustering is observed, with the Journal of Pharmacology and Experimental Therapeutics emerging as the most recurrent journal, followed by the European Journal of Pharmacology and Frontiers in Pharmacology. These are high-impact, multidisciplinary journals that tend to have a wider biological impact and reach a larger audience than the more clinically focused Web of Science discoveries. Nevertheless, across all three databases, pharmacology-focused journals remain important.

The Google Scholar database’s spread emerges from multiple independent neuroscience research institutions in the United States and Europe. The tighter concentration in Scopus suggests that foundational mechanistic studies and early agonist research were developed in parallel across different labs. This broader distribution aligns with the project’s findings that much of the field’s strength lies in preclinical glutamate and excitotoxicity research. However, these bibliometric patterns should not be seen as evidence of translational success, since they primarily exhibit publication and citation patterns, not clinical efficacy.

Prominent senior authors were identified based on influence and citation frequency. In Web of Science, Maurice (Maurice and Lockhart, 1997) contributed extensively to understanding σ1R chaperone function and its psychiatric disorder connections. Su (Hayashi and Su, 2004) played a major role in defining the receptor’s neuroprotective mechanisms. Chen and Zheng (Chen et al., 2006; Zheng, 2009) specialized in neurodegenerative modeling in preclinical models, while Jiang and Smith (Jiang et al., 2006) focused on pain modulation.

Analysis of senior authorship within Google Scholar shows a relatively scattered leadership structure, with only a small number of senior authors appearing more than once. Su emerges as the most recurrent senior author, appearing three times in the dataset, and five additional senior authors, JM Brimson, X Navarro, Maurice, Kenji, and SB Smith, each appear twice. Due to the limited recurrence of senior authors within the dataset, σ1R research is suggested to be a spread of authors from multiple independent laboratories instead of being dominated by a single centralized research group. This pattern illustrates a collaborative structure but does not necessitate interpretations of research proficiency.

With 1,446 citations, Su is the most well-known senior author according to the Scopus data. This dominance is reflected strongly in Web of Science and somewhat in Google Scholar. While writers like E. J. Cobos, with 412 citations, represents a specialized European cluster focused on pain modulation, Maurice from the University of Montpellier, with 845 citations, demonstrates a strong concentration on behavioral pharmacology and neuroprotection. This is consistent with Web of Science where these particular authors anchor the fundamental literature. They maintain high influence across the datasets, emphasizing the role of a flagship author in the development and advancement of σ1 receptor research regardless of a scattered or centralized authorship distribution. However, citation impact reflects academic influence, which is not linearly associated with the approval of translational efficacy.

As further elucidated in Figure 4, co-authorship network analysis in R identified six major research clusters with a glutamate-focused orientation in Web of Science. These clusters, largely delineated by thematic specialization and the regional collaboration of authors on σ1R, included (1) chaperones and psychiatry, (2) pain and ion channels, (3) neurodegeneration and Parkinson’s disease, (4) ligand docking, (5) medicinal chemistry, and (6) inflammation and astrocyte research. Despite the presence of these clusters, there was limited overlap between groups, indicating siloed approaches to σ1R study (Borroto-Escuela et al., 2017; Kourrich et al., 2012). Unfortunately, this was increasingly evident in translational neuroprotection research. This does not indicate failure of the field, however; structural segregation between mechanistic domains is a primary limitation to translational continuity.

Specifically, the chaperone and psychiatry-based cluster focused on molecular and behavioral outcomes related to disorders (Maurice et al., 2001). The pain and ion channel cluster explored receptor modulation of nociceptive pathways (Bangaru et al., 2013; Herrera et al., 2008; Kim et al., 2008). The neurodegeneration and Parkinson’s cluster investigated σ1R roles in neurodegenerative disease models, especially Alzheimer’s (Mancuso et al., 2012; Maurice et al., 2019; Peviani et al., 2014). Ligand docking clusters centered on computational pharmacology and drug-receptor interactions. Medicinal chemistry emphasized small-molecule development to achieve receptor specificity (Köhler et al., 2012), while the inflammation and astrocyte cluster explored glial cell contributions to excitotoxicity (Wang et al., 2015; Wang et al., 2019). Across these groups, few studies combined mechanistic research with actual clinical application. With that being said, clinical implications should be analyzed carefully, given the limited integration across the experimental and clinical categories.

On the other hand, the co-authorship network derived from the Google Scholar publications also demonstrates recurring research partnerships and institutional collaboration hubs. But compared to the Web of Science network, the Google Scholar co-authorship structure appears more expansive, likely reflecting broader indexing coverage and inclusion of interdisciplinary publications. Such connectivity would better facilitate translational research progression from molecular mechanisms to clinical applications.

Finally, the network based on Scopus data shows a clear hierarchical structure that is quite different from the scattered pattern seen in Google Scholar. The most prominent feature is a large central cluster surrounding Su and Hayashi, with connections between authors much stronger than those in the Google Scholar analysis. Beyond the central leadership group, the network reveals distinct clusters that seem to represent specific research topics or geographic regions similar to that of Web of Science. For example, Maurice’s group focusing on behavioral outcomes and psychiatry is still visible here, although a conclusion cannot be drawn on evidence strength based on this collaborative influence.

The final dataset of 300 articles met all PICO criteria, as the studies used rodent or neuronal models exposed to glutamate-induced excitotoxicity (“population”) and evaluated σ1R agonists such as PRE-084, SA4503, pridopidine, or neurosteroids (Intervention). Comparators included untreated excitotoxic controls, σ1R antagonists, or alternative neuroprotective agents (Comparator). Outcomes consistently assessed neuronal survival, calcium regulation, mitochondrial stability, oxidative stress, and behavioral recovery (Outcomes). This pattern confirms that the research questions outlined in the introduction were fully addressed through the included literature.

### 3.2 Temporal and model-based trends

Publication activity peaked between 2005 and 2015 which went hand and hand with further exploration of σ1R in various disorder-based models (Francardo et al., 2014; Ruscher et al., 2011; Shen et al., 2008). With σ1R once being a niche receptor with unclear functions, more recent research clarified its mechanistic roles across neurodegenerative and injury models, explaining the steady rise in publications and major hotspots during the 2010s. σ1R research grew rapidly and became increasingly interdisciplinary in the 25 years leading up to 2015 (Romero and Portillo-Salido, 2019). In relation to Alzheimer’s pathology, Jin et al. (2015) specifically examined σ1R involvement in modulating amyloid-related dysfunction and cognitive decline. Reviews of σ1R involvement in neurodegenerative diseases consistently show that σ1R contributes to both neuroprotection and neurorestoration, meaning it protects neurons from damage and supports their recovery afterward (Ruscher and Wieloch, 2015). After 2015, there was a notable decline in studies addressing these mechanisms, suggesting shifts in contemporary research priorities. However, this decline should not be interpreted as reduced biological importance.

Early clinical trials demonstrated modulation of σ1R-glutamate pathways but yielded mixed outcomes in cognition and motor function, likely due to the complex interplay of glutamatergic and dopaminergic signaling in human patients (Maurice, 2016; Squitieri et al., 2015). Similar studies also describe how excitotoxic depolarization events during ischemia highlight the vulnerability of neurovascular function under glutamate overload, though these relationships remain incompletely delineated in clinical translation (Shin et al., 2005).

**FIGURE 5:**
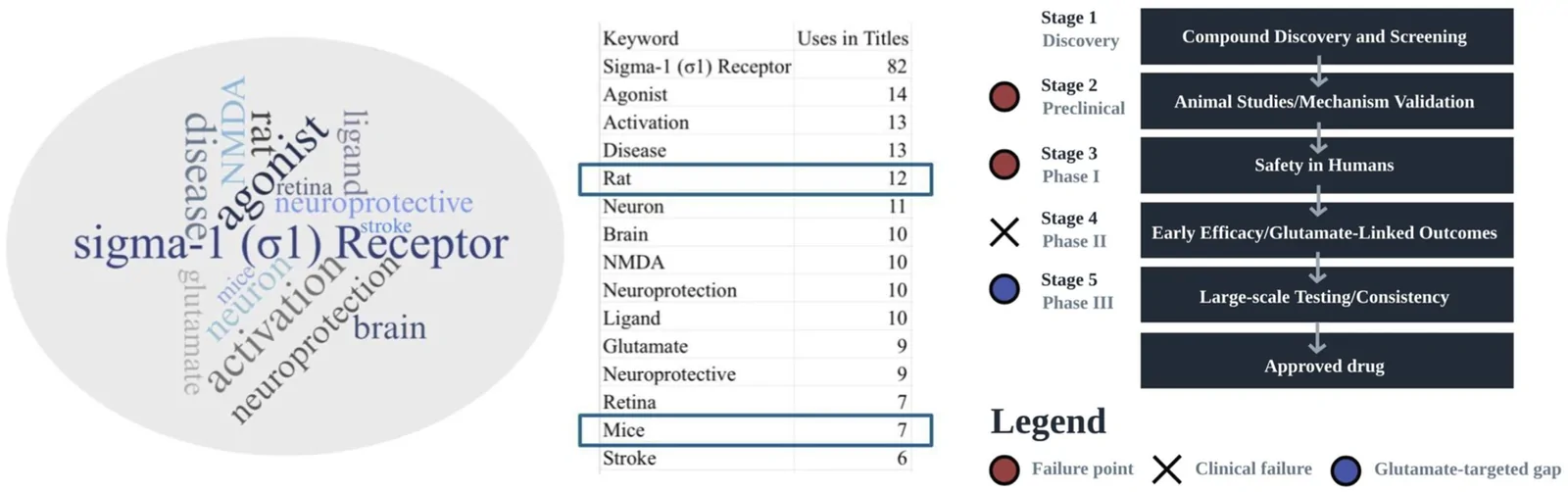
Top 15 most common keywords in titles of top 100 cited articles; barriers to drug development pipeline for o1R agonists.

An overreliance on preclinical models is also reflected in keyword trends. “Rat” and “mice” were among the 15 most frequent terms in titles of analyzed articles, with “rat” appearing as the fifth most common term and “mice” ranking 14th, as shown in Figure 5. This reinforces that most σ1R studies remain preclinical, partly because poor cellular connectivity in human neural networks complicates translational efficacy and off-target effects remain incompletely understood. Terms associated with glutamate toxicity and receptor pharmacology dominated the dataset, whereas keywords related to robust clinical trials were rare. This imbalance underscores the need for mechanistic insights to be validated in human-relevant systems. Preclinical models aside, *in vitro* systems consistently demonstrated pronounced cytoprotective effects, while *in vivo* models reflected additional complexity arising from network-level interactions (Bucolo et al., 2006; Hashimoto, 2015; Kurata et al., 2004; Senda et al., 1998; Sourbron et al., 2017; Zhang et al., 2022). These clinical chokepoints have hindered drug-development pipelines for σ1R agonists and pose major barriers to glutamate-toxicity-targeted therapies.

At the network level, several mechanistic uncertainties continue to limit the predictability of σ1R-based therapies. The receptor’s downstream effects on nitric oxide signaling and epigenetic modulation remain incompletely characterized, as do its specific interactions with astrocyte populations that play a central role in glutamate buffering (Hundt et al., 1998; Toru et al., 2001; Vagnerova et al., 2006; Zhang et al., 2019; Zheng, 2009). These mechanistic gaps are compounded clinically by off-target inflammatory responses and variability in patient neural connectivity, both of which introduce outcome heterogeneity that cannot be accounted for by receptor pharmacology alone, and which together constitute significant barriers to the development of reliable glutamate toxicity therapies. Consequently, the current research supports the hypothesis more strongly than it supports conclusive new findings.

### 3.3 Mechanisms of action

#### 3.3.1 σ1R modulation of glutamate receptor signaling and excitotoxic cascades

σ1R activation helps stabilize neuronal activity during glutamate overload by regulating NMDA receptor function and limiting there being excessive calcium entering. After ligand binding, σ1R relocates from the ER to the membrane sites where it limits the calcium influx that normally triggers mitochondrial stress and oxidative injury (Hayashi et al., 1995). This early intervention prevents the cascade of ROS production and apoptotic signaling associated with excitotoxicity (Dong et al., 2007). In multiple models, σ1R agonists reduce neuronal loss and improve tissue outcomes, demonstrating that this receptor acts upstream of major damage pathways (Allahtavakoli and Jarrott, 2011; Katnik et al., 2006; Klette et al., 1997; Nakazawa et al., 1998). These findings highlight σ1R as a key regulator of glutamate-driven injury. Glutamate toxicity is known to overload NMDA and AMPA receptors, producing excessive calcium influx that initiates excitotoxic neuronal damage (Anderson et al., 2005; Dong et al., 2007). This overload damages mitochondria by increasing reactive oxygen species and triggers cell death pathways (Anderson et al., 2005; Dong et al., 2007).

Multiple studies have examined the impact of σ1 receptor ligands on glutamate-induced excitotoxicity and calcium dysregulation across various neural systems (Guitart et al., 2000; Hirata et al., 2011; Jones et al., 2021; Jin et al., 2015; Marrazzo et al., 2005; Maurice et al., 2002; Meyer et al., 2002; Nakazawa et al., 1998; Noda et al., 2001; Ruscher et al., 2011; Ruscher and Wieloch, 2015; Shen et al., 2008; Tuerxun et al., 2010). σ1 receptors have been shown to modulate ionotropic glutamate receptor subunits, particularly NMDA and AMPA receptors, affecting excitatory neurotransmission and calcium influx, and protecting primary neurons (Bermack and Debonnel, 2005; Gonzalez-Alvear and Werling, 1994; Guitart et al., 2000; Kawamura et al., 2000; Kim et al., 2008; Meyer et al., 2002; Mohan et al., 2014; Noda et al., 2000; Noda et al., 2001; Shin et al., 2005; Urani et al., 2002). *In vitro* and *in vivo* models have demonstrated that preferential σ1 receptor ligands can attenuate glutamate-mediated cytotoxicity in cortical and hippocampal neurons (Brimson et al., 2018; Bruna and Velasco, 2018; Dun et al., 2007; Guzmán-Lenis et al., 2009; Hayashi and Su, 2007; Hong et al., 2015; Kaushal and Matsumoto, 2011; Klette et al., 1997; Luedtke et al., 2012; Nguyen et al., 2014; Rodríguez-Muñoz et al., 2015; Smith et al., 2010; Su et al., 2016; Volti and Murabito, 2016; Zampieri et al., 2023).

Earlier studies on blocked brain-blood flow injuries, also known as ischemia, showed that drugs activating the σ1R ligands like PPBP can significantly reduce the affected areas brain volume and improve neuronal survival when blood flow returns (Hong et al., 2015; Izumi et al., 2000; Klette et al., 1997; Shin et al., 2005). Other investigations document protection against ischemic damage following stroke and attenuation of retinal degeneration in ocular injury models (Bucolo and Drago, 2004; Mysona et al., 2017; Ruscher et al., 2011).

Drugs like dimemorfan and PRE-084 shrink infarct size and boost anti-inflammatory responses by reducing pro-inflammatory cytokines (Allahtavakoli and Jarrott, 2011; Shen et al., 2008. Early work also shows that σ1R plays a role in blood-flow responses and helps prevent deficits, which indirectly reduces glutamate-related injury (Shin et al., 2005). There is more evidence to suggest that natural based tryptamines, like DMT, can assist cell protection by activating σ1R (Frecska et al., 2013). DMT can reduce infarct size and improve recovery after ischemic injury, again supporting σ1R’s potential in cerebral and neuronal protection (Nardai et al., 2020). Conversely, blocking σ1 receptors can exacerbate excitotoxic cell death in neuronal cultures (Bermack and Debonnel, 2005; Borbély et al., 2022; Smith et al., 2010; Urani et al., 2002). Particularly, clinical research on dose-dependent effects of PRE-084 on retinal cells show a strong proportional relationship between dosage and cell survival, and an inversely proportional relation to DNA damage (Allahtavakoli and Jarrott, 2011; Antonini et al., 2011; Aren et al., 2011; Eddings et al., 2019; Kulkarni and Dhir, 2008; Li et al., 2010; Malar et al., 2023; Mancuso et al., 2012; Zhao et al., 2017). This happens partly through NRF2-dependent antioxidant pathways and targeted delivery to σ1R-expressing cells (Bucolo et al., 2006; Wang et al., 2016; Zhao et al., 2017).

Generally, the evidence reviewed suggests that the neuroprotective effects observed in glutamate-induced neurotoxicity models are primarily associated with σ1R agonists rather than σ1R ligands in general. Although many studies investigated compounds classified broadly as σ1R ligands, only agonists consistently activated the receptor and produced downstream effects such as reduced calcium overload, decreased oxidative stress, preservation of mitochondrial function, and improved neuronal survival. In contrast, σ1R antagonists or ligands without agonist activity generally failed to demonstrate these protective effects and were more commonly used to confirm that neuroprotection depended specifically on σ1R activation. Therefore, the findings synthesized in this review indicate that receptor activation, rather than receptor binding alone, is the critical mechanism underlying protection against glutamate-induced excitotoxicity.

**FIGURE 6:**
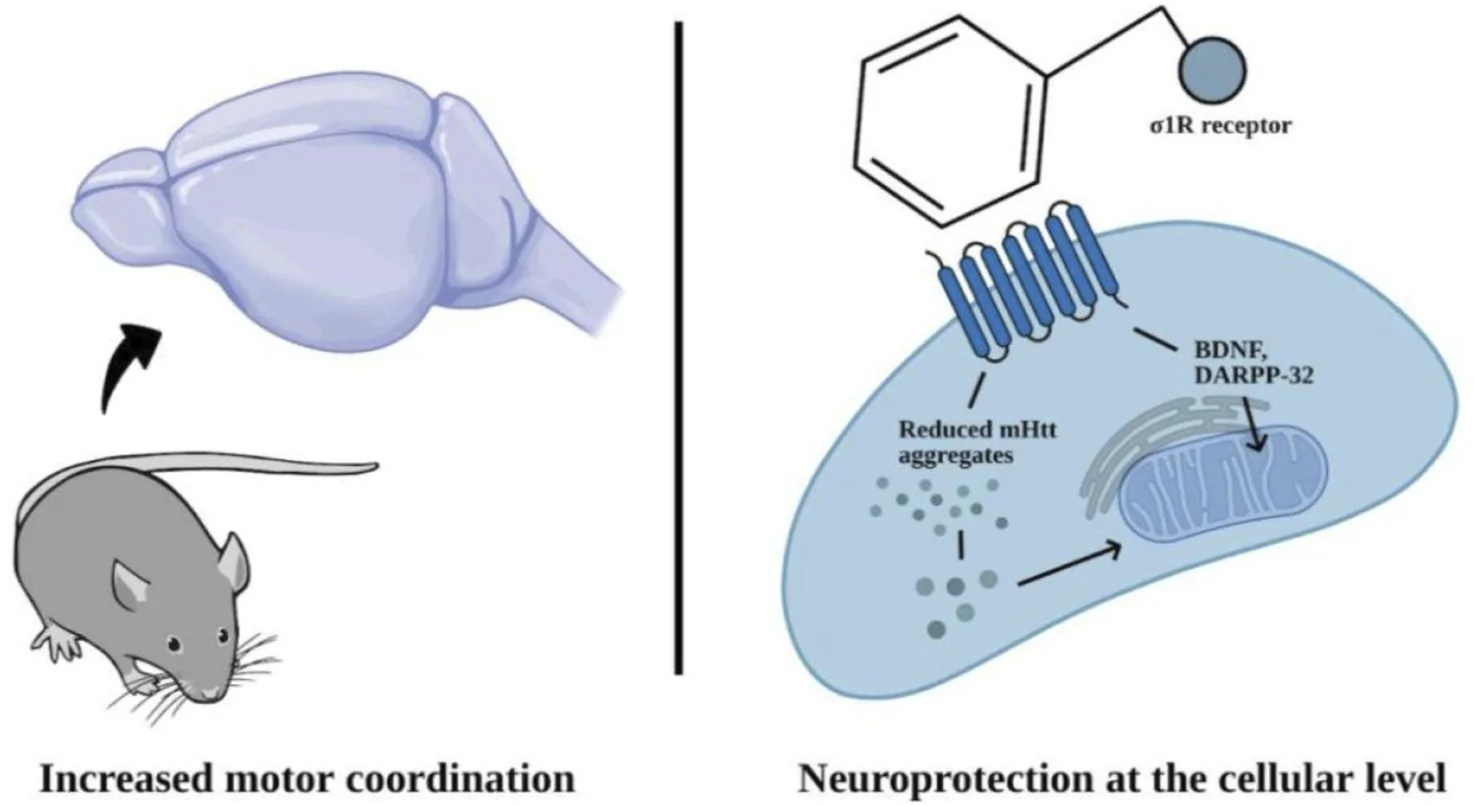
Pridopidine mechanisms in Huntington's disease. NIAID Visual & Medical Arts. (10/7/2024). Mouse Brain. NIAID NIH BIOART Source (bioart.niaid.nih.gov/bioart/369); NIAID Visual & Medical Arts. (10/7/2024). Lab Mouse. NIAID NIH BIOART Source (bioart.niaid.nih.gov/bioart/280); NIAID Visual & Medical Arts. (5/13/2025). Receptor Protein. NIAID NIH BIOART Source (bioart.niaid.nih.gov/bioart/651); NIAID Visual & Medical Arts. (10/7/2024). Mitochondria. NIAID NIH BIOART Source (bioart.niaid.nih.gov/bioart/352); Endoplasmic_Reticulum by jaiganesh via Bioicons.com, licensed under CC0.

A key example is the use of agonists in Huntington’s disease models, illustrated in Figure 6, where pridopidine provides neuroprotection and functional improvement through σ1R, helping restore calcium regulation and trophic signaling (Eddings et al., 2019; Ryskamp et al., 2017). Pridopidine enhances motor coordination by acting as a selective agonist. By activating σ1R-expressing, it restores cellular calcium homeostasis and promotes the clearance of toxic mHtt (mutant huntingtin) aggregates; this upregulates the expression and axonal transport of BDNF, boosting DARPP-32 signaling and protecting medium spiny neurons from disease-driven degeneration. Multiple studies confirm pridopidine’s positive effects that depend on σ1R activation; it has progressed to clinical trials for Huntington’s (Ryskamp et al., 2017; Squitieri et al., 2015).

σ1R agonists with stronger binding affinity and blood-brain barrier penetration tend to be more associated with higher neuroprotective scores. On net, PRE-084 and SA-4503 have higher values across multiple properties, indicating more comprehensive neuroprotection, while others show moderate effects (Allahtavakoli and Jarrott, 2011; Guo et al., 2013; Maurice, 2001; Senda et al., 1998; Motawe et al., 2020).

**FIGURE 7:**
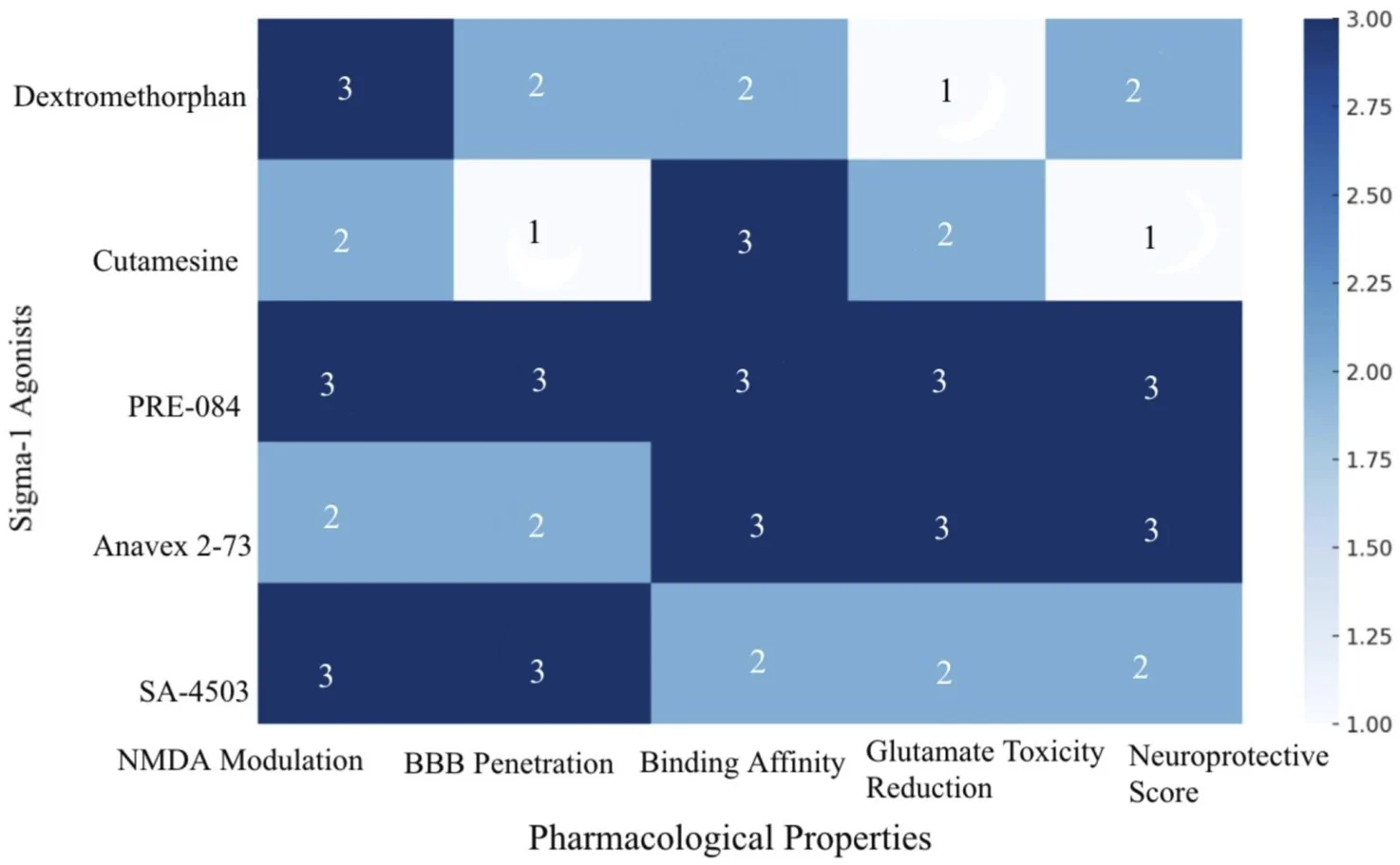
σ1R agonist properties versus neuroprotective outcomes, with a relativity scale of 1-3 sourced from the literature base.

σ1R compounds show neuroprotective or neurorestorative effects across models of stroke, ALS, Parkinson’s disease, Alzheimer’s disease, retinal degeneration, and Huntington’s disease, reported in Figure 7 (Antonini et al., 2011; Eddings et al., 2019; Francardo et al., 2014; Mancuso et al., 2012; Mavlyutov et al., 2015; Peviani et al., 2014; Piechal et al., 2021; Ruscher et al., 2011; Zhang et al., 2019).

In Alzheimer’s and related models, σ1R agonists or positive modulators have also been shown to reduce amyloid-related memory deficits and support both cholinergic and glutamatergic function (Francardo et al., 2019; Geva et al., 2021; Herrando-Grabulosa et al., 2021; Mancuso and Navarro, 2017; Maurice and Lockhart, 1997; Maurice, 2016; Mavlyutov et al., 2017; Ngo et al., 2024; Prasanth et al., 2021; Shi et al., 2022; Yamashita et al., 2015; Zhang et al., 2012; Zhao et al., 2024). In ALS mice, σ1R agonists can improve motor function, increase motor neuron survival, and modulate glial activity (Gaja-Capdevila et al., 2021; Mancuso et al., 2012; Peviani et al., 2014). In Parkinsonian models, σ1R stimulation increases neuroplasticity and motor recovery, with pridopidine and PRE-084 enhancing dopaminergic signaling and behavioral performance (Francardo et al., 2014; Francardo et al., 2019; Wang et al., 2021).

Nevertheless, many agonists failed to translate due to low receptor specificity, mixed results that involve toxicity, or inconsistent pharmacodynamics. Current strategies aim to design dual-target ligands with improved receptor binding and reduced off-target effects, though no σ1R-based therapy has yet been approved for neurodegenerative diseases (Dun et al., 2007; Franchini et al., 2020; Hayashi and Su, 2007; Hong et al., 2015; Maurice and Goguadze, 2017; Urani et al., 2002).

#### 3.3.2 σ1R regulation of ER-mitochondria calcium transfer and MAM functions

σ1R is localized at mitochondria-associated ER membranes (MAM), where it helps control Ca^2+^ transfer between the ER and mitochondria by stabilizing inositol 1,4,5-trisphosphate receptor type 3 (IP3R3) channels (Hayashi and Su, 2007; Hayashi et al., 1995; Monnet et al., 2003). When a ligand binds to σ1R, it separates from BiP and shifts into an active state that supports IP3R3 and makes ER-to-mitochondria calcium signaling longer. σ1R also supports a controlled calcium flow so that mitochondrial ATP is produced while still preventing calcium overload and permeability transition (Hayashi and Su, 2007). This ER-mitochondria coupling process is needed for energy production and cell survival (Chen et al., 2024; Couly et al., 2020; Delprat et al., 2019; Hayashi and Su, 2007; Katnik et al., 2006; Keshavarz et al., 2020; Kraskovskaya and Bezprozvanny, 2021; Lachance et al., 2023; Monnet et al., 2003; Monnet and Maurice, 2006; Maurice, 2021; Naia et al., 2021; Penke et al., 2018; Shi et al., 2021; Tchedre et al., 2008; Tchedre and Yorio, 2008; Vela, 2020; Voronin et al., 2023; Weng et al., 2017). The above stated mechanism adapts to cellular conditions, meaning that under normal activity, σ1R supports signaling, but under stress it limits calcium entry to protect mitochondrial integrity (Goguadze et al., 2017). By preserving energy balance and preventing apoptotic triggers, σ1R plays a large role in strengthening neuronal resilience during challenges that are metabolic. Having a chaperone role is one of the most consistent protective mechanisms that is known for σ1R. Agonists also make sure no harmful calcium buildup in the cytoskeleton of the cell occurs (Gonzalez-Alvear and Werling, 1995; Gronier and Debonnel, 1999; Mohan et al., 2014).

Beyond its structural role at the MAM, σ1R ligands have been shown to regulate intracellular calcium homeostasis through endoplasmic-reticulum signaling and mitochondrial protective pathways, with behavioral and electrophysiological evidence linking σ1R activity to glutamate-dependent cognitive and affective outcomes (Bermack and Debonnel, 2005; Franchini et al., 2020; Koriauli et al., 2015; Liniciano et al., 2023; Luedtke et al., 2012; Maurice and Goguadze, 2017; Pabba and Sibille, 2015; Skuza and Rogóz, 2006).

σ1R functions as a context-dependent regulator of intracellular Ca^2+^ signaling, amplifying calcium responses under physiological conditions to support processes such as long-term potentiation, while attenuating excessive Ca^2+^ influx under excitotoxic stress to prevent mitochondrial overload and apoptotic cascade activation (Goguadze et al., 2017; Monnet, 2005; Palacios et al., 2004; Su and Hayashi, 2001; Zheng, 2009). This bidirectionality is particularly evident under glutamate-induced excitotoxic conditions, where σ1R activation shifted from a modulatory to a protective role, dampening pathological calcium influx rather than amplifying it (Hirata et al., 2011; Tuerxun et al., 2010). Dextromethorphan uses neuroprotective efforts through σ1R agonism and NMDA receptor antagonism, but its effectiveness is limited by comprehensive first-pass hepatic metabolism via CYP2D6, which converts it into the less active metabolite dextrorphan, reducing CNS bioavailability. Quinidine improves neuroprotection indirectly by inhibiting CYP2D6, preventing the metabolic breakdown and increasing systemic and central nervous system concentrations of dextromethorphan, allowing for greater and prolonged σ1R engagement and NMDA receptor modulation at therapeutic levels. Recognizing this context-dependence is essential for interpreting apparently contradictory findings across experimental models, where baseline stress levels determine the direction of σ1R’s calcium regulatory effect. Thus, ligand-dependent regulation of σ1R chaperone activity is a central determinant of neuroprotective efficacy, as the receptor’s ability to stabilize IP3R3 channels and sustain ER-to-mitochondria calcium transfer is contingent on the shift induced by ligand binding (Fujimoto et al., 2012; Hayashi and Su, 2007). This same chaperone axis is engaged by endogenous neuroactive steroids, which exert a significant portion of their modulatory effects through σ1R-dependent mechanisms rather than exclusively through classical nuclear receptor pathways (Maurice et al., 2006). Both synthetic ligands and endogenous steroids achieve neuroprotection through a shared molecular bottleneck, the activation state of the σ1R chaperone complex.

Beyond calcium transfer, σ1R maintains the structural integrity of the MAM interface, in addition to antioxidant pathway involvement (Penke et al., 2018; Kinoshita et al., 2012; Klouz et al., 2008; Li et al., 2006; Shioda et al., 2012; Weng et al., 2017; Wu et al., 2021). σ1R is positioned as an intracellular protein relevant to organelle coupling, with early receptor characterization and binding-defining work providing the foundation for later mechanistic models (Alonso et al., 2000; Hanner et al., 1996; Mei and Pasternak, 2001; Monnet et al., 2003; Monnet et al., 2006).

Mechanistically, these effects contribute to increased neuronal survival and improved cognitive function in preclinical models (Inserra, 2018; Kimura et al., 2013; Moriguchi et al., 2011; Yang et al., 2012). Pathway analyses also suggest involvement of BDNF, GDNF, and BCL-2 signaling in agonists (Hashimoto, 2015; Mtchedlishvili and Kapur, 2003; Penas et al., 2011; Yang et al., 2007; Zhang et al., 2022). σ1R activating drugs help preserve the levels of BCL-2 from dropping when the cell is under stressful conditions. Since BCL-2 is a protein that protects cells from apoptosis, this helps neurons survive (Yang et al., 2007).

#### 3.3.3 σ1R’s role in dopaminergic and limbic circuit plasticity in addiction and behavior

Beyond classical neurodegeneration, σ1R is also involved in addiction and psychiatric disorders. σ1R ligands influence dopamine signaling in reward-related pathways (Borroto;Escuela et al., 2017; Maurice et al., 2002). σ1R does this by regulating presynaptic calcium channels and dopamine transporter trafficking, which both play a role in shaping dopamine release patterns. These changes affect motivation and behavioral responses to drugs, which makes σ1R a key regulator of addiction related plasticity (Gonzalez-Alvear and Werling, 1995). σ1R activation can reduce stimulant-driven behaviors and normalize stress-sensitive signaling through interactions with D2R-σ1R complexes (Borroto;Escuela et al., 2017). Beyond addiction, σ1R also contributes to mood and cognitive regulation through its effects on glutamate and serotonin pathways. These actions together can explain why σ1R ligands show broad behavioral and psychiatric connections. During cocaine exposure, σ1R complexes increase in the nucleus accumbens shell; σ1R antagonists can reduce cocaine toxicity and self-administration, affecting conditioned place preference and relapse-like behaviors (Borroto;Escuela et al., 2017; Gardner et al., 2004; Gonzalez and Werling, 1997; Martin-Fardon et al., 2007; Maurice et al., 2002; Romieu et al., 2000; Romieu et al., 2002; Romieu et al. 2004; Stefanski et al., 2004). Behavioral responses to psychostimulants were also modified through σ1R activity, including locomotor sensitization (Katz et al., 2011; Kitanaka et al., 2009; Feltmann et al., 2018).

Additional evidence showed modulation of opioid and alcohol-related behaviors and withdrawal-associated responses (Fritz et al., 2011; Moison et al., 2003; Phan et al., 2002; Vento et al., 2020; Sorger et al., 2008; Zamanillo et al., 2000). Antidepressant-like effects, particularly a reduction in despair-like behavior, were observed in rodent models following receptor activation (Bermack and Debonnel, 2005; Cabral, 2006; Francardo et al., 2019; Goodnick and Goldstein, 1998; Maurice and Romieu, 2004; Sanchez and Meier, 1997; Skuza and Rogóz, 2003). These effects were observed following both acute and chronic administration and occurred in the absence of significant changes in body weight or gross metabolic parameters. Cognitive and memory-related behavioral improvements were reported in learning paradigms and amnesia models (Ault et al., 1998; Egashira et al., 2007; Lever et al., 2016; Maurice and Lockhart, 1997; Maurice et al., 2001; Meunier and Maurice, 2004; Meunier et al., 2006; Minabe et al., 1999; Miyatake et al., 2004; Villard et al., 2011; Ukai et al., 1999).

Changes in neuronal survival signaling and dopaminergic activity were reported (Hayashi et al., 2011; Hashimoto, 2009; Ishikawa and Hashimoto, 2009; Ji et al., 2016; Ji et al., 2017; Lever et al., 2016; Martin et al., 2021; Meunier and Maurice, 2004; Villard et al., 2011; Xu et al., 2015). More recent work further linked σ1R signaling to psychiatric-relevant cellular pathways and behavioral phenotypes (Ahmed et al., 2025; An et al., 2025; Denaro et al., 2024b; Denaro et al., 2024a; Ren et al., 2025; Xiao et al., 2025).

Clinical drugs such as SSRIs and donepezil also engage σ1R, which help to explain these cognitive and mood-related actions (Cobos et al., 2008; Hashimoto, 2015). Meunier et al. (2006) showed that Donepezil also had neuroprotective effects that relied largely on activating the σ1R rather than directly binding to NMDA receptors. Through σ1R activation, Donepezil can strongly influence how NMDA receptors modulate glutamatergic signaling, helping prevent harmful overactivation and navigate the complexity of ligand binding.

#### 3.3.4 Control of retinal and visual pathway homeostasis under excitotoxic and ischemic stress

In the retina, σ1R supports neuronal survival by regulating calcium entry and oxidative stress in retinal ganglion cells (RGCs) and Müller glia (Maurice and Lockhart, 1997; Moriguchi et al., 2011). σ1R-mediated preservation of the ER-mitochondrial calcium homeostasis helps maintain photoreceptors and the retinal ganglion cell structural integrity by preventing calcium-dependent mitochondrial dysfunction, supporting the overall retinal structure under stressful conditions, the mechanistic specifics of which have been elaborated on previously. Activation of σ1R reduces glutamate induced toxicity and protects against ischemic injury by preventing calcium overload and strengthening antioxidant defenses (Bucolo and Drago, 2004). These effects are rather protective and extend to models of optic nerve damage and photoreceptor degeneration that show how σ1R agonists preserve structural integrity and visual function (Geva et al., 2021). σ1R also modulates inflammatory responses in retinal glia, which further contributes to tissue protection. Experimental studies demonstrate that overall, σ1R activation tends to have protective effects for retinal neurons (Chen et al., 2006; Kimura et al., 2013; Yang et al., 2012). In models of retinal degeneration and diabetic retinopathy, σ1R agonists preserved retinal structure, and improved electrophysiological responses (Fujimoto et al., 2012; Mysona et al., 2017; Zhao et al., 2022).

σ1R activation has been closely related to NRF2-mediated antioxidant signaling (Geva et al., 2021; Wang et al., 2025; Xu et al., 2015). Targeted σ1R ligands showed preservation of RGC survival following optic nerve injury and attenuation of photoreceptor loss in light-induced damage models (Mavlyutov and Guo, 2017; Xiao et al., 2020; Zhang et al., 2011). σ1R stimulation modulates inflammatory responses in Müller glia (Bucolo and Drago, 2004; Chen et al., 2023; Matsuno et al., 1997; Meyer et al., 2002; Xu et al., 2022).

#### 3.3.5 Endogenous neurostereoid activation of σ1R and feedback regulation of cellular stress pathways

A substantial body of work demonstrates that endogenous neurosteroids bind to and modulate σ1R activity (Maurice et al., 1998; Maurice et al., 1999; Maurice et al., 2001). These neurosteroids like DHEA and progesterone can naturally activate σ1R and start up its chaperone functions at the ER-mitochondria and can enhance synaptic plasticity, cholinergic modulation, and memory through σ1R dependent regulation of glutamatergic signaling (Kurata et al., 2004; Li et al., 2006; Noda et al., 2000; Phan et al., 2002; Ren et al., 2004). This activation can reduce ER stress, stabilize calcium signaling, and engage anti-apoptotic pathways like BCL-2 to support cell survival (Charalampopoulos et al., 2004). These findings show that σ1R integrates steroid signaling with cellular stress responses.

The examined research showed that hormonal fluctuations across aging and stress states may also influence σ1R activity, which links endocrine changes to neuronal vulnerability (Maggio et al., 2014). Hormonal regulation studies suggest σ1R activity may fluctuate with endogenous steroid levels, relating receptor function to stress responses and aging-related hormonal decline (Frecska et al., 2013; Maggio et al., 2014). Charalampopoulos et al. (2004), Charalampopoulos et al. (2006) demonstrated steroid-mediated neuroprotection through σ1R-associated signaling cascades, implicating cross-talk with growth factor pathways. Additional findings show that σ1R-dependent steroid effects can modulate cognitive performance (Maurice et al., 2006; Moebius et al., 2003; Zheng, 2009).

#### 3.3.6 Neurotrophic dependent signalling and synaptic plasticity

Studies examining neurotrophic signaling reveal that σ1R activation promotes neurite outgrowth and supports synaptic protein expression (Espallergues et al., 2007; Ruiz-Cantero et al., 2023; Tsai et al., 2009). When σ1R has interactions with BDNF, GDNF, and CREB-related signaling, it can promote dendritic spine formation, neurite extension, and synaptic strengthening (Espallergues et al., 2007). Based on experimental data, σ1R ligands increase dendritic spine density and facilitate synaptic repair following injury (Solntseva et al., 2014; Wang et al., 2015; Wang et al., 2016). These effects contribute to improved recovery after injury and help maintain structural stability in vulnerable areas. σ1R also supports axonal regeneration and cytoskeletal organization, which shows its role in neuronal maintenance (Kinoshita et al., 2012). It can be seen that σ1R activation also plays a role in enhancing neurotrophic pathways that support neuronal growth, repair, and plasticity that is long term.

In cortical models, σ1R activation modulated CREB phosphorylation and downstream gene transcription associated with synaptic strengthening (Bucolo et al., 2006; Jiang et al., 2006; Wang et al., 2019). Additional findings suggest σ1R involvement in axonal regeneration and cytoskeletal stabilization (Kinoshita et al., 2012; Srivats et al., 2016). Monnet et al. (2006) adds that σ1R participates in long-term potentiation-related mechanisms. σ1R also heavily regulates inflammatory-mediated synaptic remodeling and plasticity under chronic stress conditions (Ahmed et al., 2025; Ren et al., 2025).

#### 3.3.7 Clinical translation, off-target effects and pharmacological limitations of σ1R ligands

Despite promising preclinical findings, clinical translation has been limited (Nguyen et al., 2017; Ryskamp et al., 2019). Although σ1R ligands does show strong preclinical benefits so far, there are many compounds that interact with additional targets like NMDA receptors, α7 nicotinic receptors, and monoaminergic systems (Rodríguez-Muñoz et al., 2015; Rodríguez-Muñoz et al., 2016; Rodríguez-Muñoz et al., 2018; Sánchez-Blázquez et al., 2014). Some drugs, including dextromethorphan and cannabidiol, show σ1R-dependent neuroprotection but also act on multiple pathways, making specificity a challenge once more (Taylor et al., 2016). These off-target actions make it complicated to interpret therapeutic effects and also contribute to inconsistent outcomes across studies. Limited brain penetration and variable pharmacokinetics further hinder clinical translation. Advances in PET imaging and docking tools are improving target validation, but no σ1R-based therapy has yet reached approval (Elsinga et al., 2002).

As a result, the literature consistently frames σ1R pharmacology as polyfunctional and difficult to isolate due to multimodal pharmacological factors (Barrett, 2015; Collina et al., 2013; Davis, 2015; Kim and Maher, 2017; Kornhuber et al., 1995; Mori et al., 2001; Ryan-Moro et al., 1996; Ujike et al., 1996). This theme is explicitly defined around ligand promiscuity, cross-talk, modulation, pharmacokinetics and translational limitations (Drewes et al., 2025; Eskandari et al., 2025; Hayashi and Su, 2005; Maher et al., 2018; Moebius et al., 1996; Nguyen et al., 2014; Oyer et al., 2019; Pan et al., 2021; Rossino et al., 2020; Rousseaux and Greene, 2015; Shokr et al., 2024; Tsai et al., 2014; Kawamura et al., 2007; Matsuno and Mita, 1998; Moebius et al., 1996; Ujike et al., 1996).

As mentioned, many compounds interact not only with σ1R but also with NMDA receptors, α7 nicotinic acetylcholine receptors, monoaminergic systems, or voltage-gated Ca^2+^ channels (Cobos et al., 2008; Rodríguez-Muñoz et al., 2015; Sánchez;Blázquez et al., 2014). Further research on NMDA receptors shows that ethanol can bind to and interact with NMDA complexes in ways that make these receptors more vulnerable to excitotoxic damage (Hundt et al., 1998). σ1R agonists indirectly modulated NMDA receptor-mediated calcium entry, either by altering receptor trafficking or regulating receptor phosphorylation states and downstream calcium-buffering systems (Kim et al., 2008; Kitaichi et al., 2000; Mohan et al., 2014).

Dextromethorphan, in particular, was characterized as a low-affinity NMDA receptor antagonist and calcium channel modulator (Taylor et al., 2016; Werling et al., 2007). When used in conjunction with quinidine, it improves neuroprotection significantly due to high CNS bioavailability. In preclinical models, these combined actions were associated with robust neuroprotection across paradigms (Maurice et al., 2006; Nardai et al., 2020; Yasushi et al., 2015). Cannabidiol’s ability to reduce NMDA-mediated excitability and seizure activity is also mediated through σ1R, but with broader regulatory influence (María Rodríguez;Muñoz et al., 2018).

Many candidate σ1R-active small molecules are characterized primarily *in vitro*, while *in vivo* distribution and delivery feasibility remain major barriers (Drewes et al., 2025; Maher et al., 2018; Oyer et al., 2019; Pan et al., 2021; Rousseaux and Greene, 2015). σ1R mechanisms appear in diverse disease contexts (Brimson, 2025; Prasansuklab et al., 2025; Zhu et al., 2003). However, ligands that enhanced σ1R agonist binding in peripheral tissues, such as the liver, did not consistently reproduce these effects in brain tissue. These findings affirm that σ1R regulation operates across multiple cellular compartments and receptor systems (Fariello, 2007; Frecska et al., 2013).

**FIGURE 8:**
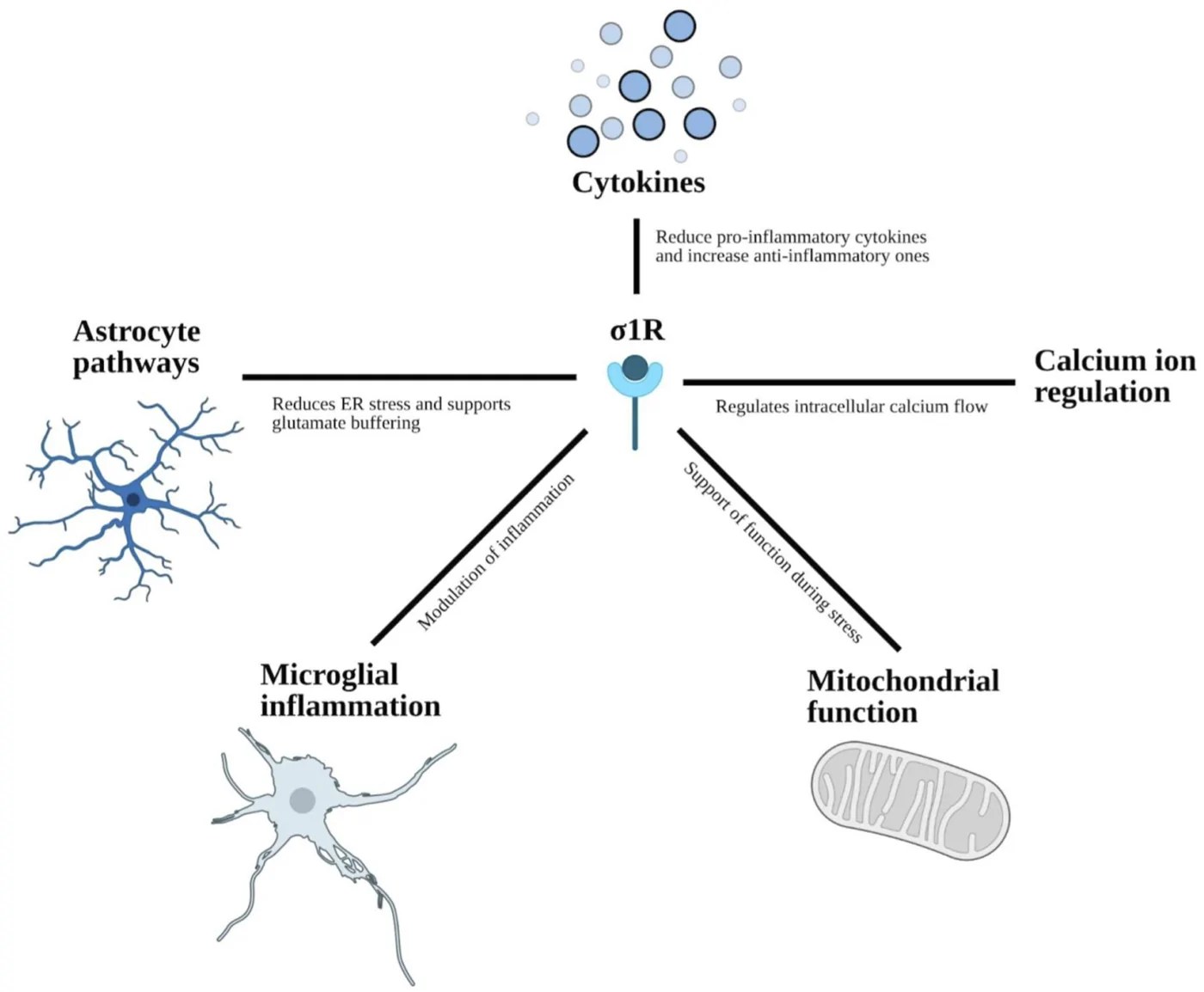
Mapping σlR signaling across neural and glial pathways. NIAID Visual & Medical Arts. (10/7/2024). Mitochondria. NIAID NIH BIOART Source (bioart.niaid.nih.gov/bioart/352); NIAID Visual & Medical Arts. (10/7/2024). Cytokines. NIAID NIH BIOART Source (bioart.niaid.nih.gov/bioart/98); NIAID Visual & Medical Arts. (10/7/2024). Astrocyte. NIAID NIH BIOART Source (bioart.niaid.nih.gov/bioart/40); NIAID Visual & Medical Arts. (10/7/2024). Langerhans Cell. NIAID NIH BIOART Source (bioart.niaid.nih.gov/bioart/285); Simple_receptor_and_ligand by Helicase 11 via Bioicons.com, licensed under CC-BY SA 4.0.

On top of that, while most σ1R agonists are designed for neuronal targets, there is a significant lack of focus on glial pathways, especially in terms of astrocyte inflammation and microglial cytokine signaling; this has been mapped in Figure 8 (Wang et al., 2015; Wang et al., 2016; Zhang et al., 2015; Zhang et al., 2019; Zheng, 2009). Addressing these gaps is essential for developing more effective pharmacological strategies to combat glutamate toxicity and emphasizes the need for collaboration among research clusters.

Allosteric modulation is treated as one way to potentially fine-tune σ1R effects rather than using full agonism and antagonism. For example, σ1R positive allosteric modulators (PAMs) enhance calcium responses and improve behavioral performance, although a narrow structure-activity window and stereochemistry can heavily shape effects and side-effect risk (Guo et al., 2013; Vavers et al., 2015). Varying effects were confirmed through saturation binding assays and dissociation kinetics analyses, which show increased agonist affinity and prolonged receptor engagement.

Translation-focused infrastructure involves ligand design or prediction tools and PET or radioligand development methods. The former involves a validated docking approach where predicted ligand efficiency correlates with experimentally measured affinity metrics (Rossino et al., 2020), while the latter is used to quantify σ-target engagement (Elsinga et al., 2002; Kawamura et al., 1999; Kawamura et al., 2005). Overall, these results show major research patterns and gaps that shape the most capital-intensive landscape of σ1R-focused neuroprotection.

## 4 Discussion

### 4.1 Bibliometric and temporal interpretations

Across decades of research, σ1R has emerged as a multifaceted chaperone. Drugs in development consistently demonstrate the receptor’s ability to modulate excitotoxic stress, supporting the hypothesis that σ1R-mediated protection is mechanistically able to support effective neuroprotective schemes across neurodegenerative and neuropsychiatric contexts (Hayashi and Su, 2004; Maurice and Lockhart, 1997; Maurice and Su, 2009; Hashimoto, 2015; Nguyen et al., 2015; Ryskamp et al., 2019).

Bibliometric and co-authorship analyses provide insight into structural challenges within the field (Romero and Portillo-Salido, 2019). Six distinct research cultures suggest existing expertise, but limited cross-cluster integration suggests siloed lines of inquiry (Chen et al., 2006; Jiang et al., 2006; Zheng, 2009). For example, translational neuroprotection efforts remain largely disconnected from medicinal chemistry or ligand docking efforts, slowing the development of ligands that could possess both mechanistic precision and therapeutic viability. Similarly, regional specialization provides for opportunities for cross-national collaboration that are currently underutilized. Enhancing collaborative bridges and reducing the top-heavy nature of the field could accelerate model refinement with diversifying methodology and perspective. Temporal trends indicating a decline in disease-specific investigation post-2015 may reflect the influence of early-stage clinical failures of candidate compounds. This trend shows the need for renewed focus on translationally relevant models.

### 4.2 Preclinical challenges

Preclinical success has not led to consistent clinical efficacy, and our findings suggest several contributing factors (Nguyen et al., 2017; Ruscher and Wieloch, 2015). Limitations of σ1R agonists for primary diseases affected by glutamate toxicity and neuroprotection have also been presented in Table 1 **.**

**TABLE 1:** Evidence and pharmacological limitations of σ1R agonists.

Pathological context	Supporting preclinical evidence	Pharmacological and translational limitations
Alzheimer’s disease	σ1R agonists show potential in reducing brain tissue damage and increasing cognitive function. They modulate amyloid-related dysfunction and counteract memory impairments, acting cooperatively with treatments like donepezil	Studies addressing relevant neuropsychiatric and neurodegenerative diseases are noticeably lacking beyond 2015. There are mixed outcomes in human cognitive translation due to the complex and unmitigated interplay of glutamatergic and dopaminergic signaling in clinical patients
Huntington’s disease	σ1R activation, specifically via the agonist pridopidine, rescues mitochondrial function and mitigates excitotoxic calcium dysregulation in disease models	Although there is clear multi-functional stabilization upstream, drug development experiences preclinical failure points for human safety due to lack of systematic validation beyond preclinical models
Amyotrophic lateral sclerosis (ALS) and Parkinson’s disease	σ1R chaperones act at the ER-mitochondria interface to handle chronic pathological stress and prevent progressive cellular collapse	Structural segregation between mechanistic domains siloes neurodegeneration research away from medicinal chemistry and docking refinement due to lack of cross-disciplinary integration
Ischemia and stroke, acute injury	σ1R reduces cortical and hippocampal tissue damage following severe ischemic events. Administering neurosteroids like DHEA improves localized memory deficits following simulated stroke	Excitotoxic depolarization events disrupt neurovascular functions during glutamate overload; these complex network relationships remain mostly unmapped. σ1R agonists function as stress stabilizers but do not directly inhibit the first acute glutamate surge on their own
General excitotoxicity and epilepsy	σ1R agonists restore calcium homeostasis and block NMDA-receptor overstimulation to prevent subsequent oxidative injury	Rapid peripheral metabolism limits sustained drug levels within mammalian brain tissues. Overreliance on preclinical rodent keywords shows a distinct lack of validation in human neural systems
Retinal systems	σ1R agonists promote retinal ganglion cell protection and attenuate photoreceptor loss, limiting glial inflammatory responses	Translational blocks occur due to cross-reactivity and lack of absolute structural specificity for σ1R over secondary targets, such as NMDA.

The complexity of σ1R ligands cuts both ways, their ability to act on multiple pathways can boost protective effects, but it makes it harder to determine which outcomes are truly σ1R-dependent. Interactions with NMDA or monoaminergic receptors can introduce psychiatric or cognitive side effects, and species differences in brain circuits and receptor expression likely explain why benefits observed in rodents do not consistently translate to humans. These gaps highlight the need for ligands with greater selectivity, particularly for MAM-specific σ1R pools.

Second, ligand specificity remains a critical barrier. As stated above, many σ1R agonists engage multiple targets, introducing off-target effects that reduce clinical predictability (Cobos et al., 2008; Guo et al., 2013; Hayashi and Su, 2004). As a result, although σ1R agonists show benefits across many popular neurological diseases, the type of benefit they provide depends mainly on the pathology. In ALS and other motor neuron diseases, σ1R activation mainly protects vulnerable neurons and shapes glial responses. In Parkinson’s models, σ1R ligands tend to promote neurorestoration and synaptic repair (Siddiqui and Bhatt, 2023). In Alzheimer’s models, they more strongly reduce cognitive and synaptic deficits. Seeing this variation confirms that σ1R is not acting through one fixed pathway and instead functions as a context-dependent molecule whose effects shift according to the dominant stressors in each disease. σ1R-targeted drugs may need to be tailored to each condition, with ligand selection and dosing used mainly for the most relevant downstream pathways, rather than assuming a single, universal neuroprotective mechanism.

σ1R has also been explored in psychiatric and addiction contexts, where it functions not only as a cellular stress regulator but as a modulator of circuit-level plasticity (Maurice et al., 2002). Both agonists and antagonists show utility depending on whether the therapeutic goal is to reduce harmful plasticity or reinforce adaptive responses. This bidirectionality means that compounds designed to block excitotoxicity may also influence reward and motivational circuits, adding a layer of complexity to neuroprotective drug development.

Third, human variability in neuronal connectivity and astrocytic signaling, along with mixed results during the transitional window, may complicate therapeutic consistency, because biological context is as influential as receptor pharmacology. σ1R agonist efficacy varied across experimental models, with robust protection observed in acute excitotoxic and ischemic paradigms and more variable outcomes in chronic neurodegenerative models (Cai et al., 2008; Izumi et al., 2000; Schetz et al., 2007; Shin et al., 2005; Szabó et al., 2021). Differences in glial involvement, which is largely underexplored when it comes to specific mechanistic consequences, likely contributed to this variability. As astrocytes and microglia increasingly appear central to neurodegenerative progress, addressing ambiguities regarding downstream nitric oxide signaling and epigenetic modulation in glial populations is important for developing therapeutics that operate effectively within human cellular environments.

Despite this variability, σ1R activation consistently stabilized calcium signaling and stress responses at the ER-mitochondria interface. Steroid-based agonists tend to produce broader metabolic and antioxidant effects, while small molecule agonists like PRE-084 and SA4503 act more directly on MAM-related calcium control. σ1R can also translocate away from the MAM to interact with ion channels and kinases elsewhere in the cell, suggesting a broader connectivity role linking organelle stress responses to larger-scale neuronal signaling changes. Better characterizing this flexibility will be important for understanding and managing off-target effects.

Fourth, the biggest interpretive issue is mechanistic attribution. When a compound influences σ1R and other systems, observed outcomes can’t be confidently labeled as σ1R-dependent without stronger controls (Barrett, 2015; Collina et al., 2013; Davis, 2015; Kim et al., 2012; Kim and Maher, 2017; Kornhuber et al., 1995; Mori et al., 2001; Ryan-Moro et al., 1996). Medicinal chemistry sensitivity limits real-world robustness and safety (Vavers et al., 2015). Inclusion of docking validation work and PET development reflects a push to improve selectivity-aware design and quantify target engagement *in vivo*, both of which are prerequisites for reducing false positives from polypharmacology (Elsinga et al., 2002; Kawamura et al., 1999; Kawamura et al., 2005; Kawamura et al., 2007; Matsuno and Mita, 1998; Rossino et al., 2020; Ujike et al., 1996). Translation likely requires either better selectivity or better context stratification rather than assuming one σ1R drug will behave uniformly across disease settings (Brimson, 2025; Danthi et al., 2006; Prasansuklab et al., 2025; Zhu et al., 2003).

### 4.3 Drug applications

σ1R activation functions primarily as a “stabilizer” of cellular stress response, so to speak, rather than having a direct connection to early excitatory neurotransmitter release (Hayashi and Su, 2007). Since σ1R agonists protect well against later injury but don’t fully control the first glutamate surge, they likely cannot stop acute excitotoxic damage on their own, especially with their high reactivity (Franchini et al., 2020). Findings suggest that they would need to be paired with drugs that block early glutamate release or excessive NMDA receptor activity.

σ1R agonists also demonstrated rapid peripheral metabolism, limiting sustained brain concentrations despite strong preclinical efficacy. In these cases, reduced neuroprotection correlated with insufficient central drug levels. The literature consistently indicated that effective σ1R-mediated neuroprotection required adequate temporal and spatial drug availability within neural tissue. Differences in bioavailability and metabolic clearance contributed to heterogeneous outcomes across disease models and dosing regimens (Maurice, 2021; Nguyen et al., 2017).

### 4.4 Study limitations

Several limitations inherent to the scoping review process should be considered when interpreting these results. By design, this review prioritized breadth over depth, which limits the ability to formally assess study quality, risk of bias, effect size magnitude, and other methods of data charting across individual investigations. These limitations show the need for more standardized methods and broader clinical validation, especially deeper mechanistic integration to strengthen future σ1R research.

The focus on the top 100 cited articles may also bias the synthesis toward earlier or more influential studies, potentially underrepresenting emerging work or less-cited but methodologically innovative contributions. In addition, heterogeneity in experimental models constrained direct comparison across studies, necessitating qualitative synthesis rather than quantitative aggregation, although bibliometric analysis offers valuable structural insight. To summarize, this most prevalent evidence and research gaps collectively shape the future direction of σ1R pharmacology and clarify where the field must advance to achieve meaningful clinical progress.

## 5 Conclusion

σ1R is unique as a therapeutic target because it can address diverse neuropathological contexts. Firstly, agonists activate neuroprotective mechanisms, preventing neuronal injury. Secondly, they regulate dopaminergic circuits and neuronal pathways, which causes a neurochemical shift helpful for combating addiction. Thirdly, σ1R plays a role in the movement of calcium ions, which can have a cascading effect on regulation of ion channels and signaling. Calcium signaling triggers cellular responses, particularly the mitochondrial energy exchange. Fourthly, antagonists inhibit σ1R function; this in conjunction with agonistic drug delivery can lead to clinically significant modulation effects in brain damage and cognitive decline.

σ1R agonists present compelling mechanistic potential in these ways, but future progress depends on resolving challenges about ligand specificity, heterogeneity, model fidelity and robustness, and research fragmentation (Nguyen et al., 2017; Maurice and Nino Goguadze, 2017). The available evidence is overwhelmingly preclinical; building on this rich foundation of knowledge, research has the potential to transform σ1R’s mechanistic insight into usable treatment for populations affected by neurodegeneration. Across the analyzed literature, several consistent concepts emerged: σ1R’s role as a ligand-regulated molecular chaperone, its involvement in calcium homeostasis at the ER-mitochondria interface, its capacity to modulate mitochondrial resilience and stress adaptation, and interactions involving other drug classes. The evidence presented in this review shows that σ1R agonists play a role in mitigating glutamate induced neurotoxicity across many experimental models, which supports their relevance as regulators of calcium homeostasis and ER-mitochondria signaling. This mechanistic connection suggests that interventions involving σ1R may offer a therapeutic approach for multiple brain diseases that have glutamatergic dysfunction as their core pathological feature.

Taken together, the findings of this scoping review provide a comprehensive overview of the conceptual landscape and evidentiary bases underlying σ1R agonism as a neuroprotective strategy, more so in the context of glutamate-induced neurotoxicity. For key stakeholder groups, including basic neuroscientists, pharmacologists, medicinal chemists, and translational researchers, these findings clarify the mechanistic strengths that justify continued interest in σ1R and the structural and methodological barriers that have constrained clinical progress. Potential next steps include prioritizing human-relevant experimental systems, developing ligands with improved specificity and brain bioavailability, incorporating astrocytic and microglial endpoints into neuroprotection models, encouraging cross-disciplinary collaboration between mechanistic and translational research clusters, and using modern technology to bridge the gap that has been evident since 2015. Addressing limitations will be essential to inform the engineering of the next-generation of receptor-targeted neurotherapeutics. σ1R remains a promising but underdeveloped therapeutic target and to advance its pharmacological potential, it will require coordinated efforts across mechanistic and clinical translation research.

## Data Availability

The original contributions presented in the study are included in the article/Supplementary Material, further inquiries can be directed to the corresponding author.

## Supplementary material

SUPPLEMENTARY TABLE S1 Exported study metadata and study characteristics from Web of Science, Google Scholar, and Scopus, with evidence gap mapping.
